# High Resolution, Annual Maps of Field Boundaries for Smallholder-Dominated Croplands at National Scales

**DOI:** 10.3389/frai.2021.744863

**Published:** 2022-02-25

**Authors:** Lyndon D. Estes, Su Ye, Lei Song, Boka Luo, J. Ronald Eastman, Zhenhua Meng, Qi Zhang, Dennis McRitchie, Stephanie R. Debats, Justus Muhando, Angeline H. Amukoa, Brian W. Kaloo, Jackson Makuru, Ben K. Mbatia, Isaac M. Muasa, Julius Mucha, Adelide M. Mugami, Judith M. Mugami, Francis W. Muinde, Fredrick M. Mwawaza, Jeff Ochieng, Charles J. Oduol, Purent Oduor, Thuo Wanjiku, Joseph G. Wanyoike, Ryan B. Avery, Kelly K. Caylor

**Affiliations:** ^1^ Graduate School of Geography, Clark University, Worcester, MA, United States; ^2^ Department of Natural Resources and the Environment, University of Connecticut, Storrs, CT, United States; ^3^ Clark Labs, Clark University, Worcester, MA, United States; ^4^ Independent Researcher, Tucson, AZ, United States; ^5^ Independent Researcher, San Francisco, CA, United States; ^6^\SpatialCollective, Nairobi, Kenya; ^7^ Department of Geography, University of California Santa Barbara, Santa Barbara, CA, United States; ^8^ Earth Research Institute, University of California Santa Barbara, Santa Barbara, CA, United States; ^9^ Bren School of Environmental Science and Management, University of California Santa Barbara, Santa Barbara, CA, United States

**Keywords:** active learning, machine learning, PlanetScope, smallholder cropland, field size, label error, Africa, Ghana

## Abstract

Mapping the characteristics of Africa’s smallholder-dominated croplands, including the sizes and numbers of fields, can provide critical insights into food security and a range of other socioeconomic and environmental concerns. However, accurately mapping these systems is difficult because there is 1) a spatial and temporal mismatch between satellite sensors and smallholder fields, and 2) a lack of high-quality labels needed to train and assess machine learning classifiers. We developed an approach designed to address these two problems, and used it to map Ghana’s croplands. To overcome the spatio-temporal mismatch, we converted daily, high resolution imagery into two cloud-free composites (the primary growing season and subsequent dry season) covering the 2018 agricultural year, providing a seasonal contrast that helps to improve classification accuracy. To address the problem of label availability, we created a platform that rigorously assesses and minimizes label error, and used it to iteratively train a Random Forests classifier with active learning, which identifies the most informative training sample based on prediction uncertainty. Minimizing label errors improved model F1 scores by up to 25%. Active learning increased F1 scores by an average of 9.1% between first and last training iterations, and 2.3% more than models trained with randomly selected labels. We used the resulting 3.7 m map of cropland probabilities within a segmentation algorithm to delineate crop field boundaries. Using an independent map reference sample (*n* = 1,207), we found that the cropland probability and field boundary maps had respective overall accuracies of 88 and 86.7%, user’s accuracies for the cropland class of 61.2 and 78.9%, and producer’s accuracies of 67.3 and 58.2%. An unbiased area estimate calculated from the map reference sample indicates that cropland covers 17.1% (15.4–18.9%) of Ghana. Using the most accurate validation labels to correct for biases in the segmented field boundaries map, we estimated that the average size and total number of field in Ghana are 1.73 ha and 1,662,281, respectively. Our results demonstrate an adaptable and transferable approach for developing annual, country-scale maps of crop field boundaries, with several features that effectively mitigate the errors inherent in remote sensing of smallholder-dominated agriculture.

## 1 Introduction

Amidst all the challenges posed by global change, a particular concern is how agricultural systems will adapt to meet humanity’s growing food demands, and the impacts that transforming and expanding food systems will have on societies, economies, and the environment (Searchinger et al., 2019). A number of efforts are underway to address various aspects of this challenge, including work on diagnosing and closing yield gaps (Lobell et al., 2009; Licker et al., 2010; Mueller et al., 2012), expanding and commercializing production (Morris and Byerlee 2009), and to understand (Rulli and D’Odorico 2014; Kehoe et al., 2017; Davis et al., 2020) and mitigate (Estes et al., 2016b) agriculture’s ecological impacts. The success of these efforts depends heavily on data that accurately describes the location and characteristics of croplands (Fritz et al., 2015), and, given the rapid pace of agricultural change (Gibbs et al., 2010; Zeng et al., 2018; Bullock et al., 2021), how these are changing from 1 year to the next. Unfortunately, for many regions, existing cropland datasets are inaccurate, and are usually created as once-off or infrequently updated products. As such, estimates of global cropland area tend to vary widely, often disagree about where croplands are located (e.g. Fritz et al., 2011, Fritz et al., 2013), and become rapidly outdated. Errors in these maps can propagate in subsequent analyses that use cropland data as inputs, resulting in potentially misleading answers (Estes et al., 2018). Beyond distributions, few data are available on key cropland characteristics such as field size, an important variable needed to estimate yield and other key food security variables (Carletto et al., 2015), and as an indicator of farm size (Levin 2006; Samberg et al., 2016), a critical component of rural livelihoods given increasing population densities and longstanding debates about the relationship between farm size and productivity (Feder, 1985; Carletto et al., 2013; Desiere and Jolliffe, 2018).

The deficit of information is due to the fact that in many regions the only source of cropland data are remotely sensed land cover maps, which are prone to error. This is particularly true in Africa (Fritz et al., 2010; Estes et al., 2018), where agricultural changes will be largest and the need for accurate baseline data is thus greatest (Searchinger et al., 2015; Estes et al., 2016b; Bullock et al., 2021), and where the characteristics of croplands exacerbate the error inherent in remote sensing analyses. Half of all fields in Africa’s smallholder-dominated agricultural systems are smaller than 1 ha (Lesiv et al., 2019). This size is small relative to the 30–250 m resolution of the sensors typically used in many landcover mapping efforts (e.g. Chen et al., 2015; Sulla-Menashe et al., 2019), which results in errors due to mixed pixels and aspects of the modifiable area unit problem (Openshaw and Taylor 1979; Boschetti et al., 2004), wherein the pixel’s shape does not match that of crop fields, and is too coarse to aggregate into an approximation of that shape (Dark and Bram 2007; Estes et al., 2018). On top of the matter of scale is the high variability within and between fields, their tendency to intergrade with surrounding vegetation (Estes et al., 2016a; Debats et al., 2016), and the high temporal variability within croplands. These last three aspects pose challenges for the classification algorithms that are applied to the imagery.

Recent technological advances are helping to overcome these challenges. Chief among these are the growing numbers of satellites that collect high (<5 m) to near-high (10 m) resolution imagery at sub-weekly intervals (Drusch et al., 2012; McCabe et al., 2017). The spatial resolution of these imagery addresses the scale mismatch between sensor and field, and their high frequency captures the seasonal dynamics of cropland, which helps classifiers distinguish cropland from surrounding cover types (Debats et al., 2016; Defourny et al., 2019). On top of this, the opening of satellite image archives (Wulder et al., 2016) and advances in cloud computing are placing large volumes of moderate to near-high resolution imagery together with the computational and algorithmic resources necessary to classify them at scale (Gorelick et al., 2017). These capabilities have already been used to create a new generation of higher resolution (10–30 m) cropland and landcover maps for Africa and other regions [ESA (n.d.); Lesiv et al. (2017); Xiong et al. (2017) (Zhang et al., 2021);]. However, the potential of the highest resolution (<5 m) imagery to map cropland over very large extents (e.g. country scales) has yet to be realized, presumably because these data are commercial and relatively expensive, and require significant computational resource to process.

Beyond the imagery and computational gains, machine learning algorithms are rapidly advancing, providing large gains in classification performance (Maxwell et al., 2018; Ma et al., 2019). However, the ability to take advantage of these gains is often limited by newer models’ need for large training datasets, which are typically unavailable, hard to collect, or contain numerous errors (Ma et al., 2019; Elmes et al., 2020; Burke et al., 2021). To build sufficient training samples, as well as the reference data needed to objectively assess their performance (we refer collectively to both types as “labels,” distinguishing between each as needed), map-makers rely heavily on visual interpretation of high resolution satellite or aerial imagery (Chen et al., 2015; Xiong et al., 2017; Stehman and Foody 2019), as it is impractical and expensive to collect these data in the field over large areas, particularly on an ongoing basis. Consequently, a number of web-based platforms have been developed to collect such labels (Fritz et al., 2012; Estes et al., 2016a; Bey et al., 2016). Image-drawn labels present two particular problems. The first is that they inevitably contain errors of interpretation, which can vary substantially according to the skill of the labeller, particularly over complex croplands with small field sizes (Estes et al., 2016a; Waldner et al., 2019). The second problem is that visual interpretation depends on high resolution imagery (<5 m), as fields are increasingly difficult to discern as image resolution decreases. Typically the only available source of high resolution imagery is “virtual globe” basemaps (e.g. Bing or Google Maps), which present mosaics of high resolution satellite and aerial images collected over a span of several years (Lesiv et al., 2018). This within-mosaic temporal variation can create a temporal mismatch between the labels and the imagery being classified, which is usually from a different source (e.g. Landsat, Sentinel; Xiong et al. (2017)). If a land change occurs in the interval between the two image sets (e.g. a new field was created), the label, even if accurately drawn, introduces error into the classifier. This source of error may be elevated in croplands where swidden agriculture is practiced (Van Vliet et al., 2013), or in rapidly developing agricultural frontiers (Zeng et al., 2018). Despite the high potential for it, label error is often not considered during model training and map accuracy assessment, resulting not only in the potential for maps to be misused or misinterpreted, but in missed opportunities to improve model performance (Estes et al., 2018; Stehman and Foody, 2019; Elmes et al., 2020).

Taking into consideration the advances and remaining limitations described above, the ability to map smallholder-dominated croplands can be further improved by 1) more fully exploiting the profusion of high frequency, high resolution imagery provided by CubeSats (McCabe et al., 2017), and 2) by implementing methods that improve the ability to collect and minimize errors in image-interpreted labels. We developed a mapping approach that focuses on these two sources of improvement. Our approach uses PlanetScope imagery collected by Planet’s fleet of Dove satellites, which provide 3–4 m resolution imagery over large areas at near daily intervals (McCabe et al., 2017; PlanetTeam 2018), at relatively low to no cost for academic research^1^ and non-commercial, sustainability-oriented applications^2^. Although these data are of lower spectral depth and, in some cases, quality, than Landsat, Sentinel, or Worldview imagery, their daily revisit enables country-to continent-scale image mosaics to be created for multiple periods during a single agricultural year, even over the cloudiest forest regions where it is hard to successfully construct cloud-free composites from optical imagery with longer return intervals (even by a few days). This ability to capture intra-annual variability can be more important for classifying cropland than spectral depth (Debats et al., 2016). Beyond the frequency, PlanetScope’s 3.7 m resolution–although substantially coarser than the 0.5–1 m imagery available in most areas covered by virtual globes–is sufficiently resolved for humans to discern small fields under many conditions (Fourie 2009; Estes et al., 2018). This allows labels to be made using the same imagery that is classified, which helps to minimize label error. To further reduce label noise, we developed a platform that includes rigorous label accuracy assessment protocols and a novel approach for creating consensus labels, which helps reduce mistakes made by individual labellers (Estes et al., 2016a; Elmes et al., 2020). We couple the labelling platform with a machine learning model inside an active learning (Cohn et al., 1994; Tuia et al., 2011) framework, in which the model is trained interactively, using the model’s prediction uncertainty over unlabelled areas to select new sites for additional labelling (Cohn et al., 1994; Tuia et al., 2011). This approach helps boost the performance of the classifier while reducing the overall number of labels required to achieve a given level of performance (Debats et al., 2017; Hamrouni et al., 2021). An unsupervised segmentation step is then applied to convert pixel-wise cropland predictions into vectorized maps of individual field boundaries.

Here we use this approach to create a high resolution, national map of crop field boundaries in Ghana, a country where smallholder farming predominates across a broad mix of climate and agricultural systems, ranging from primarily grain and vegetable crop production in the northern savannas to tree crop-dominated systems in the forested southwest, including large areas where shifting agriculture is practiced (Samberg et al., 2016; Kansanga et al., 2019). The map represents a single agricultural year (2018–2019), as opposed to a multi-year epoch, thereby demonstrating a capacity for annual, high resolution maps that can be used to monitor rapidly evolving small-scale agricultural systems, including key characteristics such as field size. In addition to providing valuable new data and insight into Ghana’s agriculture, our study demonstrates one of the most spatially extensive agricultural applications of CubeSats to date, provides a new technique for converting daily imagery into seasonal composites, and shows how best practices for model training and label collection can be applied to improve map accuracy (Elmes et al., 2020).

## 2 Materials and Methods

**FIGURE 1:**
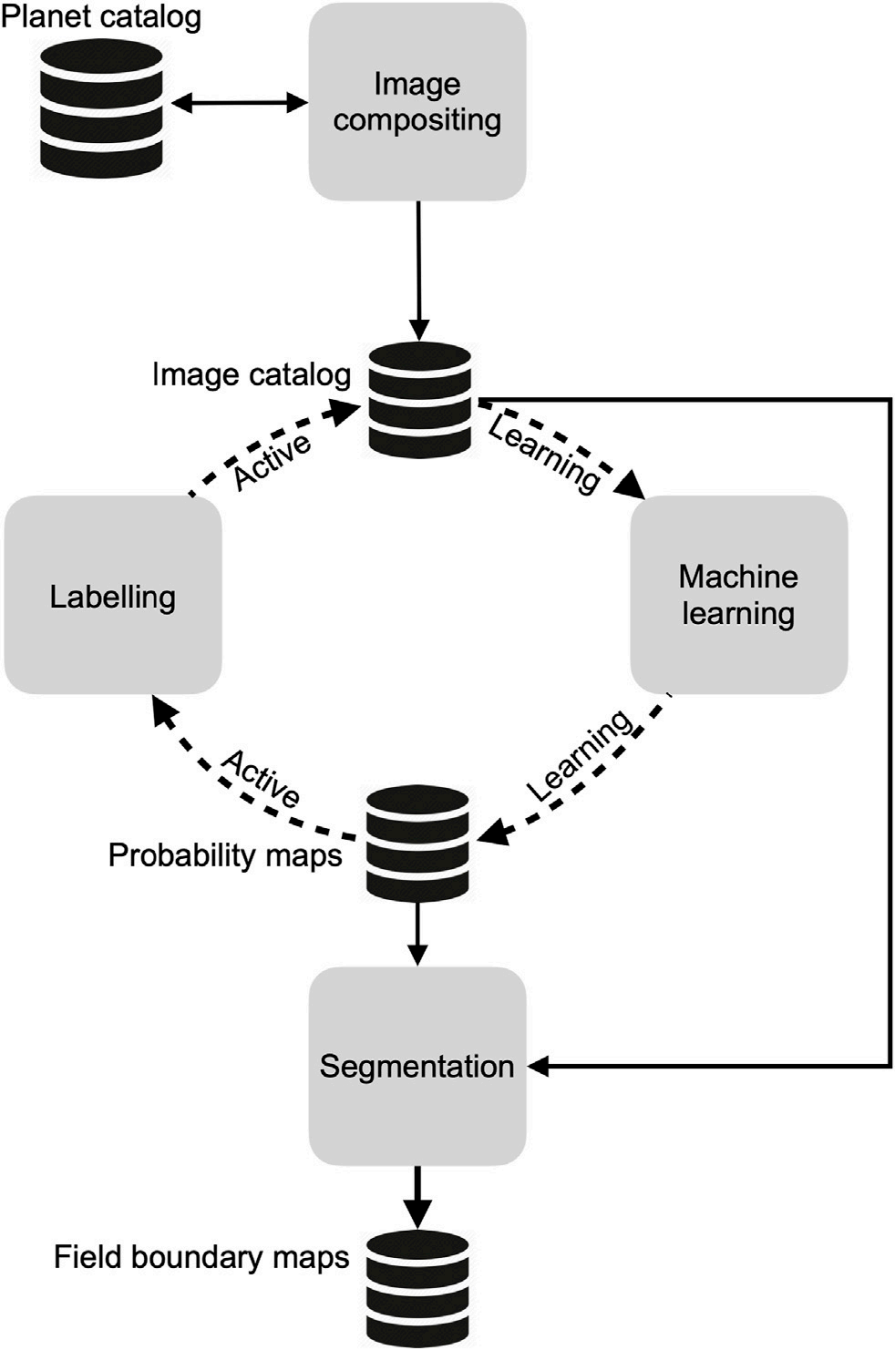
An overview of the primary mapping components, the data stores that hold the inputs and outputs from each component, and the direction of connections between them. The dashed line indicates iterative interactions, while solid lines indicate one-time or irregular connections.

The mapping approach we developed is comprised of four open source components (Figure 1) that are designed to run in a cloud computing environment. The first component collects daily PlanetScope imagery and converts them into cloud-free seasonal composites. The second is a custom-built platform that provides tools for labelling the composites, along with procedures to assess and minimize label error. This platform interacts with the third component, a machine learning process, within an active learning (Cohn et al., 1994; Tuia et al., 2011) loop, to produce a map of predicted cropland probabilities for each image pixel. The fourth and final component is an algorithm that segments the image composites, then filters the resulting polygons using the pixel-wise cropland predictions produced by the active learning classifier, resulting in a final set of vectorized field boundaries.

We describe each component in further detail in the following section, and how we applied them to map Ghana’s annual cropland boundaries, excluding tree crops.

### 2.1 Image Compositing

The image processing component was designed for PlanetScope Analytic surface reflectance imagery (PlanetTeam, 2018), which provides three visual (red, green, blue) and near-infrared bands at 3.7 m resolution at nominal daily frequency. The images are provided as ortho-rectified and converted to surface reflectance, although there are residual errors from inter-sensor differences and the radiometric normalization process (Houborg and McCabe, 2018), variation in the orientation of scene footprints, as well as a high frequency of cloud cover over the study region (Wilson and Jetz, 2016; Roy et al., 2021) that are not fully captured by the provided cloud masks. To minimize the effect of these residual errors, we developed a procedure for creating temporal composites of the primary growing and non-growing seasons within a single 12-month period. For Ghana, we defined the primary growing season as May through September, followed by the off (or dry) season from November or December through February. We chose these two seasons because prior work shows that the contrast between them improves cropland classifications (Debats et al., 2016), Furthermore, capturing the seasons in this sequence during the same year helps minimize differences caused by land change. The wide time intervals we used to define each season were necessary for collecting a sufficient number of images to make high quality composites, as Ghana’s cloud cover renders many scenes unusable and therefore unavailable in Planet’s catalog, thus the effective return interval can be substantially longer than 24 h during the cloudiest months (Roy et al., 2021).

We collected all available scenes intersecting Ghana and falling within these two seasons during the 2018 agricultural year (defined here as March 2018-February 2019) via the Planet API (PlanetTeam, 2018), and transferred these to cloud storage (Amazon Web Services [AWS] S3). We then converted each scene into analysis ready data (Dwyer et al., 2018) by cropping each to the boundaries of a 0.05° grid that it intersected (see Supplementary Figure S1), which provided the dimensions for making composited image tiles. We chose this cell size for tiling because it is slightly narrower than the short axis of a PlanetScope scene, which increases the number of intersecting scenes that completely cover the tile, thereby helping to minimize edge artifacts in the composites.

To create a seasonal composite, we calculated two weights for the time series of each pixel within the ARD stack for a given season:

$${W1}_{t}=\frac{1}{{blue}_{t}^{2}}$$ (1)

$${W2}_{t}=\left\{\begin{array}{cc} \frac{1}{{NIR}_{t}^{4}},\; & \text{if}{NIR}_{t}<median\left\{{NIR}_{\text{t}1},{NIR}_{\text{t}2},\ldots ,{NIR}_{\text{ti}}\right\}.\\ 1,\; & \text{otherwise}. \end{array}\right.$$ (2)

Where *t* is a particular date in the pixel time series, which begins at date 1 for the given compositing period and ends on date *i*, blue is the blue band, and NIR the near infrared band. Eq. (1) assigns lower weights to hazy and clouded pixels as the blue band is sensitive to these atmospheric features (Zhang et al., 2002), while Eq. (2) assigns low weights to pixels in cloud shadow (Zhu and Woodcock 2012; Qiu et al., 2020).

After assigning these two weights, we calculated the final composited pixel value:

$$\bar{B}=\frac{\sum_{t=1}^{T}B_{t}*{W1}_{t}*{W2}_{t}}{\sum_{t=1}^{T}{W1}_{t}*{W2}_{t}}$$ (3)

Which is the weighted mean for each pixel for each band *B* for the given season.

Each composited seasonal tile was saved as a cloud-optimized geotiff, and a “slippy map^3^” rendering was created for each composite using Raster Foundry (Azavea 2020), for display within the labelling platform (section 2.2.1).

We generated a catalog of 16,232 composite tiles (hereafter simply “tiles”) for Ghana, consisting of a seasonal pair for each of the 8,116 0.05° tile grid cells covering Ghana. To assess the quality of the resulting composites, 50 tile grid cells were randomly selected, and two separate observers graded each corresponding seasonal composite using four categories that evaluated the degree of 1) residual cloud and 2) cloud shadow, 3) the number of visible scene boundary artifacts, and 4) the proportion of the image with resolution degraded below the 3.7 m PlanetScope resolution (e.g. because of between-date image mis-registrations). Each category was qualitatively ranked from 0 to 3, with 0 being the lowest quality, and 3 the highest (see SI for complete protocol), making the highest possible score 12. We rescaled scores to fall between 0 and 1.

### 2.2 Mapping Cropland Probabilities With Active Learning

The first step in creating a country-wide field boundary map of Ghana was to create a pixel-wise classification of cropland probabilities throughout the country. Given the high resolution of the imagery and the need to minimize the computational burden, we divided Ghana into 16 distinct mapping regions, or Areas of Interest (AOIs). We constructed the AOIs by grouping together tile grids into blocks representing the larger 1° cells used to assign tile identifiers (Supplementary Figure S1A). We grouped tile cells from 1° degree cells that overlapped Ghana’s boundaries together with those from the nearest 1° cell contained entirely within Ghana (with the exception of AOI 16, which was comprised of tile grids from the 1° cells along Ghana’s southern coast. The average extent of the resulting AOIs was 15,457 km^2^ (range 12,160–23,535 km^2^).

We used the active learning process to develop a separate cropland classification model for each of these AOIs, based on an approach described by Debats et al. (2017). We initiated the process by training a starter model using labels from a set of randomly selected training sites drawn from a 0.005° grid that was nested within the tiling grid. This finer grid, which we refer to as the “primary grid” for simplicity, provided the target area for creating labels (section 2.2.1), as well as the unit for distributing computing jobs (section 2.2.2). We then assessed the performance of the starter model against a separate set of validation labels developed for each AOI, applied the model to predict cropland probabilities for pixels in unlabelled primary grid cells in each AOI, and calculated an uncertainty criterion (Debats et al., 2017):

$$Q_{I}=\underset{I\left(x,y\right)\epsilon I}{\sum }{\left(p\left(x,y\right)-0.5\right)}^{2}$$ (4)

Where Q is the uncertainty for each unlabelled primary grid cell I, calculated from the predicted probability *p* of a randomly selected subset of pixels (x, y) drawn from it. Pixels with predicted probabilities closer to 0.5 are least certain as to their classification, thus the lowest values of *Q* represent primary grid cells posing the most difficulty for the classifier.

We ranked the unlabelled primary grid cells from least to most certain, randomly selected a subset of cells from the top 30% of the ranking (to minimize the risk of spatial autocorrelation), and sent these back to the labelling platform. After these new sites were labelled, they were added to the starter pool of labels, the model was retrained with the larger training set, its performance and prediction uncertainty was reassessed, and a new sample of the most uncertain primary grid cells was again sent for labelling. This loop was typically repeated for 3 iterations, after which a final map of cropland probabilities was made.

In the next two sections, we describe the labelling and machine learning components of the active learning process in more detail.

#### 2.2.1 Labelling

To collect the initial randomized samples for model training, we grouped the AOIs (Supplementary Figure S1A) into three clusters based on approximate agro-ecological similarity: the 6 northernmost savanna-zone AOIs (Cluster 1), a central to southeastern cluster (Cluster 2) consisting of the 3 middle (AOIs 7–9) and 2 southeastern AOIs (12 and 15), and a southwestern cluster (Cluster 3) made up of the forest zone AOIs (10, 11, 13, 14, 16). Within each cluster, we randomly selected and labelled 500 primary grid cells, which provided relatively large initial training samples for these agro-ecologically similar regions, while helping to minimize the overall amount of labelling effort. To create validation samples, we randomly selected and labelled 100 primary grid cells per AOI, and a further 100 cells were labelled in each AOI during each active learning iteration.

In addition to training and validation labels, we also collected training reference labels and map reference labels (Elmes et al., 2020). The former were a set of 98 primary grid cells selected to represent the range of cropland types and densities in Ghana, which were labelled by expert analysts (the lead researchers on this project). We used these to assess the performance of the individual labellers collecting training and validation labels. Map reference labels were collected and used to assess the accuracy of the final map (see Section 2.4).

We collected all labels using a custom-built platform that we adapted from an earlier prototype we developed for crowdsourced labelling (Estes et al., 2016a). We enhanced this platform by making several major additions, including an independent backend that allowed us to recruit and manage our own labelling teams, improved procedures for assessing and improving label accuracy, and processes for automating the machine learning component. The platform runs on a cloud-hosted Linux virtual server (AWS EC2) and is comprised of a database (PostGIS/Postgres), a mapping interface (OpenLayers 3), an image server (Raster Foundry), and a set of utilities for managing, assessing, and converting digitized field boundaries into rasterized labels.

**FIGURE 2:**
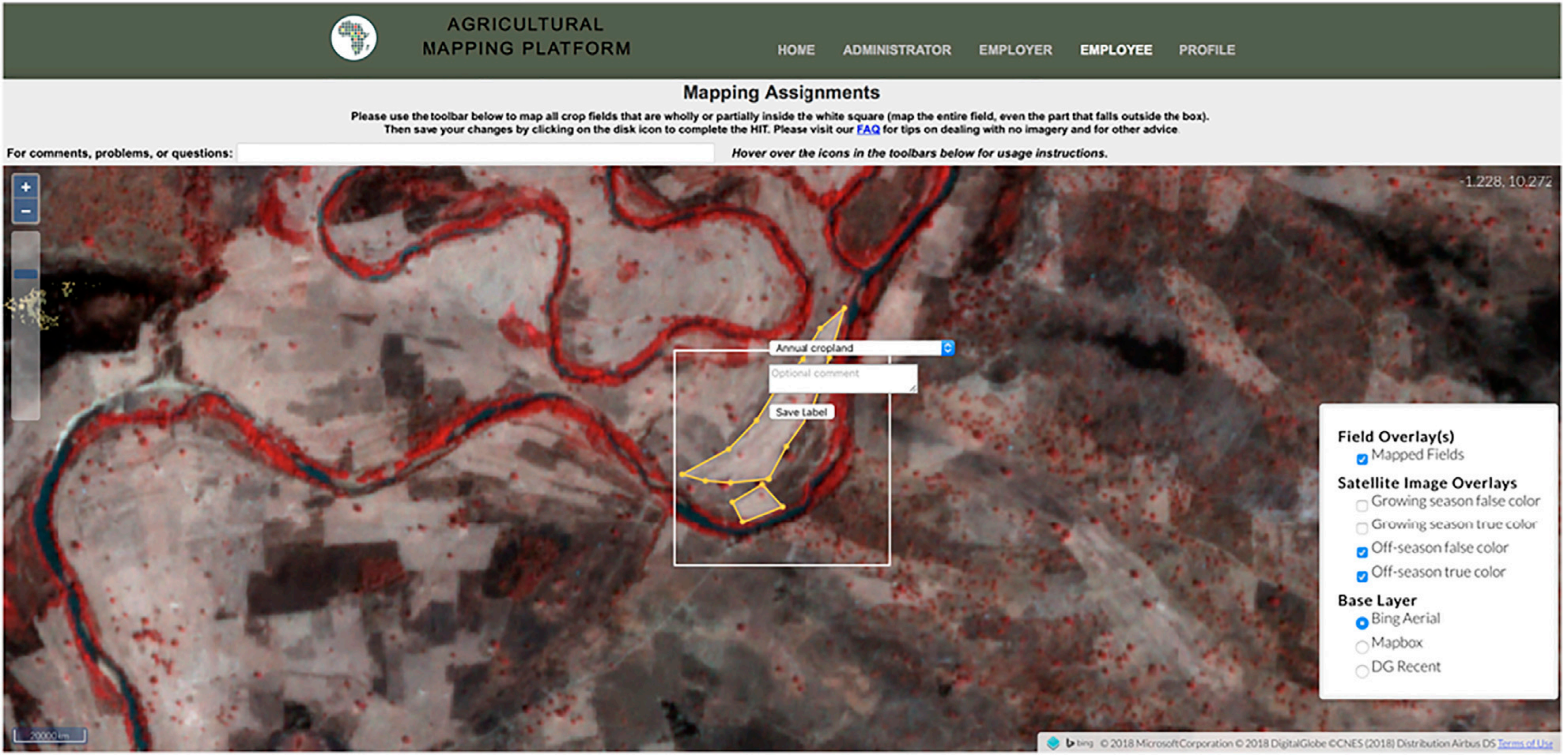
An overview of the labelling platform’s interface.

We created a separate labelling instance for each AOI. To create training and validation labels, labellers (the co-authors of this paper) logged into the website (built with Flask) for a particular AOI and navigated to the mapping interface (Figure 2), where they were presented with a white target box representing a primary grid cell to label, a set of digitizing tools, and several different sources of imagery. These included true and false color renderings of the growing season and dry season PlanetScope composites, and several virtual globe basemaps. They then used the polygon drawing tool to digitize the boundaries of all crop fields visible within the PlanetScope overlays that intersect the target grid cell. For this project, labellers were instructed to digitize active or recently active crop fields, avoiding tree crops, and fallow or potentially abandoned fields (see SI for digitizing rules). To aid with interpretation, labellers toggled between the PlanetScope renderings and the basemaps to help form a judgement about what constitutes a field. The labeller assigned each digitized polygon a class category (e.g. annual cropland), saved all completed fields to the database, and were then presented with the next target to label. If the target grid cell did not contain any fields, labellers simply pressed save to go to the next cell.

The flow of labelling targets presented to each worker was determined by the platform’s built-in scheduler. Each primary grid cell selected for labeling was placed into a queue within the platform’s database, and converted into a labelling task with a specified number of assignments (the boundaries drawn by an individual labeller) that had to be completed in order to finish the task. There were two types of tasks, accuracy assessment or model training/validation, with the assignments for each indistinguishable to labellers. Upon completing an accuracy assessment assignment, the platform invoked a scoring algorithm that compared the labeller’s digitized boundaries against a set of training reference polygons, resulting in a label quality score:

$${score}_{i}=\beta_{0}I+\beta_{1}O+\beta_{2}F+\beta_{3}E+\beta_{4}C$$ (5)

Where *i* indicates the particular assignment, and *β*_0−4_ represent varying weights that sum to 1. *I* refers to “inside the box” accuracy, *O* is the accuracy of those portions of the labeller’s polygons extending beyond the target grid boundaries, *F* is fragmentation accuracy, a measure of how many individual polygons the labeller delineated relative to the reference, *E* measures how closely each polygon’s boundary matched its corresponding reference polygon boundary, and *C* assesses the accuracy of the labeller’s thematic labels (see SI for individual formulae). Eq. (5) is an extension of the approach described by Estes et al. (2016a).

We configured the platform’s scheduler to present workers with accuracy assessment assignments at a rate of 1 for every 5 assignments mapped. This generated a history of accuracy assessment scores that we used to assess label quality and minimize label error.

For training and validation, where there was no reference data to assess label accuracy, we set each task to have four assignments, i.e. each was completed by four separate labellers. When all four assignments were complete, a Bayesian merging routine was invoked to combine the four sets of labels into a single consensus label:

$$P\left(\theta |D\right)=\overset{n}{\underset{i=1}{\sum }}P\left(W_{i}|D\right)P\left(\theta |D,W_{i}\right)$$ (6)

Where *θ* represents the true cover type of a pixel (field or not field), *D* is the label assigned to that pixel by a labeller, and *W*_*i*_ is an individual labeller. P(*θ*|D) is the probability that the actual cover type is what the labellers who mapped it says it is, while P(W_*i*_|D) is the average score (ranging between 0 and 1) of the accuracy assessment assignments an individual labeller completed within the AOI, and P(W*θ*|D, *W*_*i*_) is the labeller’s label for that pixel. This approach therefore used the average assignment quality score to weight each labeller’s label for a given pixel (see SI for further details). Each pixel in the target grid cell was merged using this approach (*n* = 40,000), which helps to minimize individual labeller’s errors. We estimated a confidence measure for each consensus label by calculating its Bayesian Risk (see SI), which ranges between 0 and 1, with 0 indicating full agreement between labellers for all pixels, and 1 indicating complete disagreement.

#### 2.2.2 Cropland Classification Model

Upon completing each batch of labels, the platform automatically launched a machine learning cluster (Elastic Map Reduce^4^) comprised of several hundred to a thousand CPUs, depending on the size of the AOI.

The first step in the process was to derive a set of features from the image composites. Previous work showed that a large number of simple features summarizing image reflectance and vegetation indices within local neighborhoods were highly effective for classifying smallholder croplands (Debats et al., 2016). We followed that logic in this study, but used a smaller feature set because the storage and memory required for our mapping geographies were several orders of magnitude larger. For each seasonal composite, we calculated the mean and standard deviation of each band within an 11 × 11 and 5 × 5 moving window, respectively (initial tests revealed these two window sizes to be most effective). This provided an overall set of 24 features, including the unmodified bands of both composites (Table 1).

**TABLE 1:** List of image features.

Feature	Window size	N Features
RGB-NIR	1 × 1	8
Mean	11 × 11	8
Standard deviation	5 × 5	8

We used a combination of GeoTrellis^5^, rasterio^6^, and RasterFrames^7^ to derive the features on the fly (which was enabled by converting the composites to Cloud-optimized Geotiffs^8^) and convert them into Apache Spark DataFrames.

The extracted features were combined with their corresponding training and validation labels and passed to the machine learning classifier, a SparkMLlib implementation of Random Forests (Breiman 2001). We trained the model with a balanced sample and a tree depth of 15 and total tree number of 60. Initial testing showed that model performance saturated with increasing values of these parameters (cluster failures occurred when tree depths and numbers were simultaneously ≥16 and ≥50 , respectively), and that model stability was satisfactory with these settings, as there was ≤0.01 difference in accuracy for separate models trained on the same labels.

#### 2.2.3 Model Performance

To assess performance of the Random Forests classifier, we used the validation sample to calculate binary accuracy, the F1 score (the geometric mean of precision and recall), and the area under the curve of the Receiver Operating Characteristic (Pontius and Si, 2014), as well as the false positive rate. We calculated these measures each time the model was retrained for a given AOI, in order to assess the change in classifier performance with each active learning iteration.

To evaluate whether active learning improved model performance relative to randomized label selection, we ran an additional test within three AOIs (1, 8, and 15), in which we retrained the model with 100 randomly selected labels for each iteration. We then compared the differences in accuracy, AUC, and F1 between the actively and randomly trained models (Debats et al., 2017).

To quantify the potential impact of label error on classification results, we conducted two further analyses. We evaluated the performance differences between models trained with three different sets of labels: 1) those from the lowest scoring labeller to map each training site, 2) those from the highest scoring labeller, and 3) the consensus labels. We also calculated the correlations between the mean Bayesian Risk of labels in each AOI and the corresponding model performance metrics (Supplementary Table S3).

### 2.3 Segmentation

Upon completion of the active learning process, we deployed a five-step algorithm to create a segmented map of field boundaries (see Supplementary Figure S3 for illustration of the steps). In the first step, we identified edge features within the imagery. To do this, we applied the meanshift algorithm (Yizong Cheng, 1995) to each dry-season composite tile, and then passed a Sobel filter over the mean-shifted green, red, and near-infrared bands, and the corresponding map of predicted cropland probabilities. We then summed the four resulting edge images to produce a combined edge image.

In the second step, we used a compact watershed algorithm (Neubert and Protzel, 2014) to segment the edge image, specifying a high number of segments (6,400) per tile, so that the mean segment size (<0.5 ha) was finer than the expected mean field size (>1 ha).

In the third step, we hierarchically merged the resulting polygons. We first constructed a region adjacency graph for each tile, with each node representing all image pixels within each polygon. The edge between two adjacent regions (polygons) was calculated as the difference between the means of the normalized colors of all bands. We then merged the most similar pairs of adjacent nodes until there were no edges remaining below the predetermined threshold of 0.05.

In the fourth step, we overlaid the merged polygons with the cropland probability images, and polygons in which the mean probability was greater than 0.5 were retained as crop fields.

In the fifth and final step, we refined the crop field polygons, by removing holes and smoothing boundaries using the Visvalingam algorithm (Visvalingam and Whyatt, 1993). We then merged neighboring polygons that overlapped along tile boundaries.

The resulting map represents dry season crop field boundaries, as we did not segment growing season images. We made this choice because labels were primarily drawn on dry season composites, when boundaries were typically more visible.

### 2.4 Map Assessment

We followed recommended guidelines (Stehman and Foody, 2019) to conduct an independent assessment of the categorical accuracy of the final maps, using a set of 1,207 (487 cropland; 720 non-cropland) point-based, map reference labels, which were placed across Ghana using a stratified random sample design, and collected through the labelling platform by two expert supervisors (see SI for full details on sample design and collection). For efficiency, the supervisors labelled separate portions of the sample, but overlapped on a small subset (*n* = 23). We calculated the label agreement (87%) on this subset to estimate uncertainty in the map reference sample (Stehman and Foody, 2019). In addition to this, the sample was labelled with four classes: cropland; non-cropland; unsure but likely cropland; unsure but likely non-cropland. The last two classes, which constituted 15.7% of the sample, provided a further measure of uncertainty in the map reference sample.

We used the sample to calculate the overall accuracy for each map, the class-wise User’s and Producer’s accuracy, and the 95% confidence intervals for each accuracy measure (Olofsson et al., 2013; Olofsson et al., 2014; Stehman and Foody, 2019). We calculated these measures across the entire country, as well as several different zones, to evaluate regional difference in accuracy. We defined two sets of zonations (Supplementary Figure S5), each containing four zones, the first created by grouping 1) the three northern AOIs (1–3), 2) the six central AOIs (4–9), 3) the four southwestern AOIs (10, 11, 13, 14, 16), and 4) the two southeastern zones (13, 15). This grouping differs from the three clusters used to collect initial model training samples, as we designed these to divide the country more finely, and to isolate the less forested southeastern third of Ghana from the more forest northwest. The second zonation was developed by grouping the country’s eight agro-ecological zones into four broader clusters (Supplementary Figure S5B). We applied this zonation only to the per-pixel classification, to better understand patterns of error in the model.

To assess how effectively the segmentations captured field characteristics, we compared the size class distributions of the segmented field boundaries against those calculated from the field boundaries digitized by the labellers within the 100 validation sites from each AOI. We chose this approach because of existing uncertainties in polygon-based accuracy assessment methods (Ye et al., 2018), and because the map’s ability to represent field sizes was of greatest interest. To undertake this comparison, we selected the polygons from the most accurate labeller to digitize the 100 validation grids in each AOI, and calculated the average area and number of polygons in each cell. We then calculated the same statistics from the segmented boundaries that intersected each validation grid, and compared the two sets of statistics.

We used the final maps to evaluate the characteristics of Ghana’s croplands. We calculated the estimated area of cropland in Ghana, as well as the average size and total number of fields in the different AOIs. We used the map reference sample to calculate adjusted area estimates and confidence intervals for each map class, and used the differences between labellers' polygons and segmented boundaries at validation sites to calculate bias-adjusted estimates of mean field sizes and the total number of fields.

## 3 Results

Our results produced two separate maps of Ghana’s annual croplands, over a total area of 248,343 km^2^ that included portions of the neighboring countries overlapped by image tiles.

### 3.1 Image Quality

**FIGURE 3:**
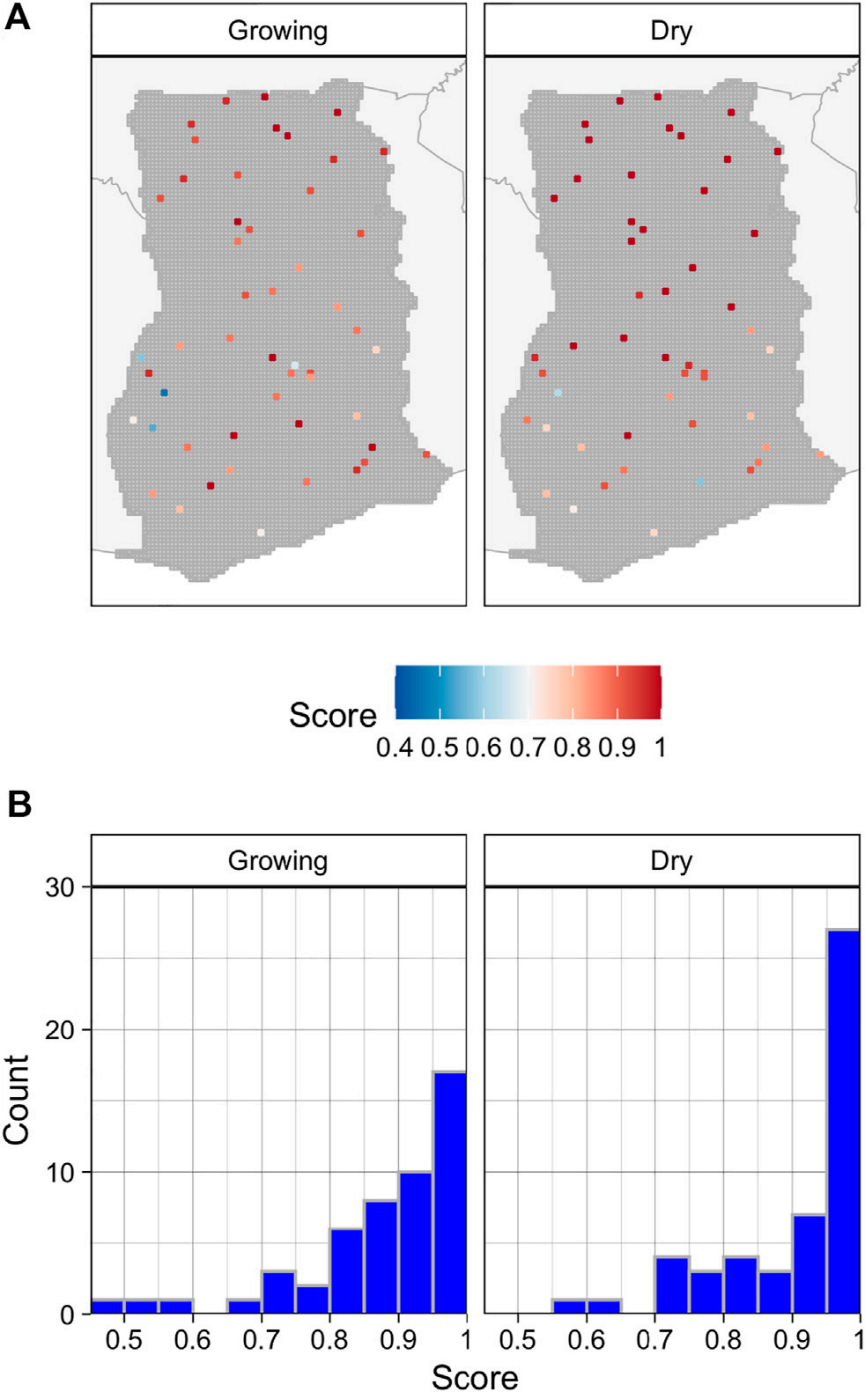
The location and quality scores of 100 randomly selected tiles **(A)** for the growing and off-growing season, and the corresponding distributions of the quality for each season **(B)**.

The assessment of image composites found that their quality in both seasons was highest in the northern half of the country and lowest in the southwest, (Figure 3A), where the substantially greater cloud cover resulted in a much lower density of available PlanetScope imagery for each time period (Supplementary Figure S6). The average quality score of growing season composites was 0.88, with 70 percent having scores ≥0.85 (out of 1; Figure 3B), while the mean score of dry season composites was 0.92 (74 percent ≥0.85).

### 3.2 Cropland Probabilities

To make the initial maps of cropland probabilities, the active learning process ran for 3 iterations in 12 of 16 AOIs, varying from as little as 1 to as many as 4 iterations across the other 4 AOIs, with the number of iterations varying according to the performance of the starter models (i.e. AOIs with higher starting performance stopped after fewer iterations). Each AOI’s model was trained by 300–500 randomly selected labels (Supplementary Figure S7A), plus an additional 100–400 (typically 300) labels within each AOI that were selected by active learning. Actively selected labels showed distinctive patterns in several AOIs (Supplementary Figure S7B), such as concentrating along ecotones or the boundaries of agro-ecological zones. A total of 6,299 training and 1,600 validation labels were collected by 20 labellers to develop and assess model performance (Supplementary Figure S8).

#### 3.2.1 Performance Gains During Active Learning

**FIGURE 4:**
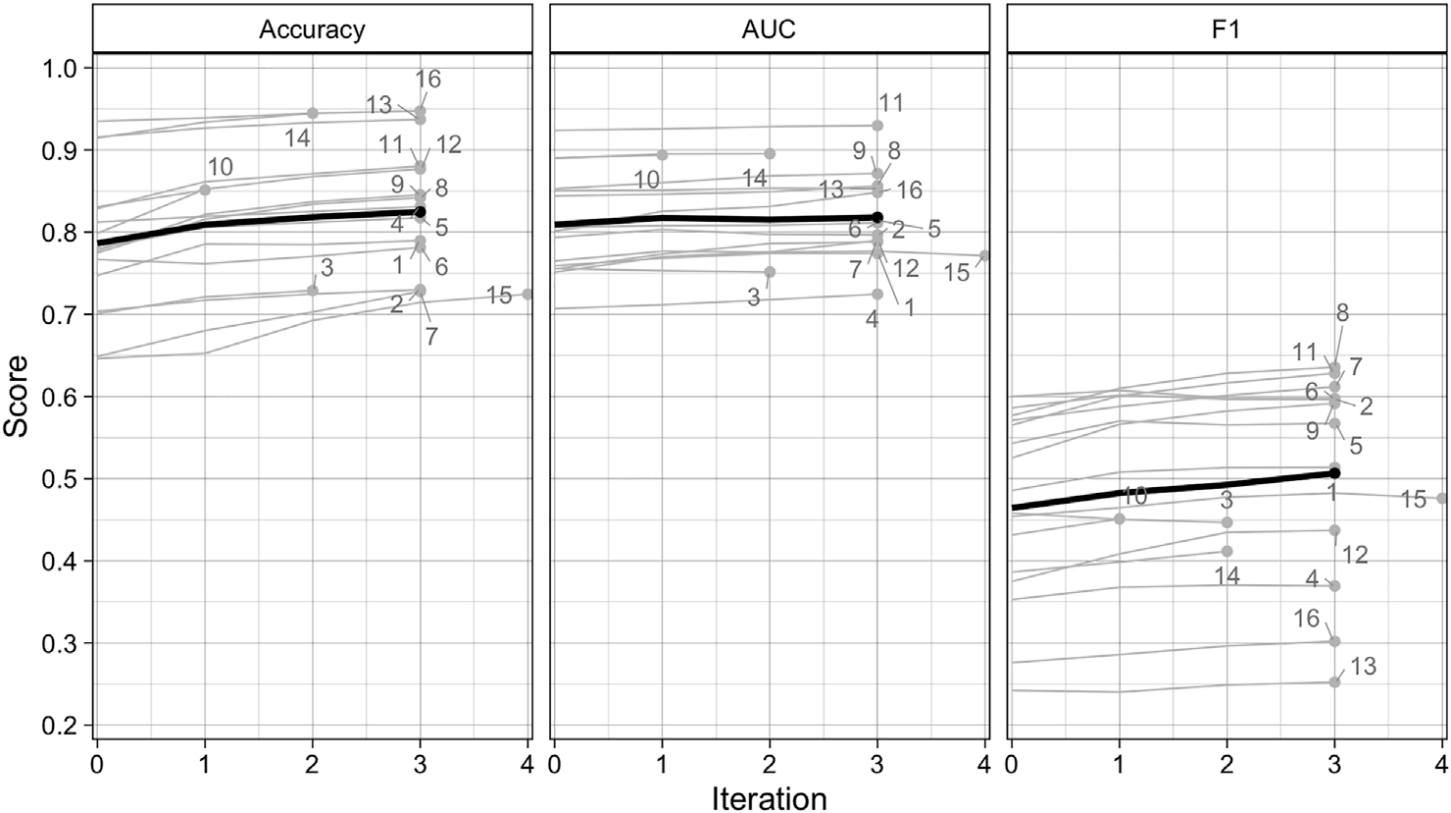
Scores for overall accuracy, area under the curve of the Receiver Operating Characteristic, and the F1 scores for the Random Forests model results after each iteration of the active learning loop for each AOI (gray lines), as well as the mean score per iteration across all AOIs (black lines).

The performance of the Random Forest classifier typically improved with each active learning iteration. The average accuracy, AUC, and F1 at iteration 0 were 0.786, 0.809, and 0.464, respectively, increasing to 0.825, 0.818, and 0.507 by iteration 3 (Figure 4). These differences represent respective gains of 4.9, 1.1, and 9.1 percent for the three metrics. The largest gains for each metric occurred on iteration 1, averaging 2.9, 1, and 3.8 percent for accuracy, AUC, and F1, while the lowest gains were realized on iteration 3, with accuracy, F1, and AUC respectively increasing by just 1.2, 0.9, and 0.3%. The scores achieved on the final iteration varied substantially across AOIs and metrics. Accuracy ranged between 0.725 (AOI 15) and 0.948 (AOI 16), while AUC varied from 0.725 (AOI 4) and 0.93 (AOI 11), and F1 from 0.252 (AOI 13) and 0.636 (AOI 8).

The experiment conducted in three AOIs (in AOIs 1, 8, and 15) showed that training models with active learning improved performance compared to randomized approaches to label selection. After three iterations, the accuracy, AUC, and F1 scores for the actively trained models were respectively 0.8, 0.6, and 2.3 percent higher than those for randomly trained models (Supplementary Figure S9). However, there was more variability in earlier iterations, with average score differences of −1.7 (accuracy), 0.6 (AUC), and 0.8 percent (F1) after iteration 1, and −0.3 (accuracy), 0.4 (AUC), and 1.8 (F1) percent after iteration 2

#### 3.2.2 The Impact of Label Error and Uncertainty on Model Performance

We used the two measures of label quality calculated by the platform, the average quality score of each labeller and Bayesian Risk (or simply “label risk”), to assess the potential impacts of label error on model performance. The average of each labeller’s AOI-specific accuracy score was 0.71 (range 0.6–0.85; see Supplementary Figures S8, S10 for details on label scores and number of assignments per labeller). The average Bayesian Risk was 0.124, with highest label risk (0.165) in the northern AOIs (AOIs 1–6; Supplementary Figures S11, S12), lowest (0.165) in the southwestern AOIs (AOIs 10, 11, 13, 14, 16), and intermediate (0.131) in the central-southeastern AOIs (AOIs 7–9, 12, 15).

**FIGURE 5:**
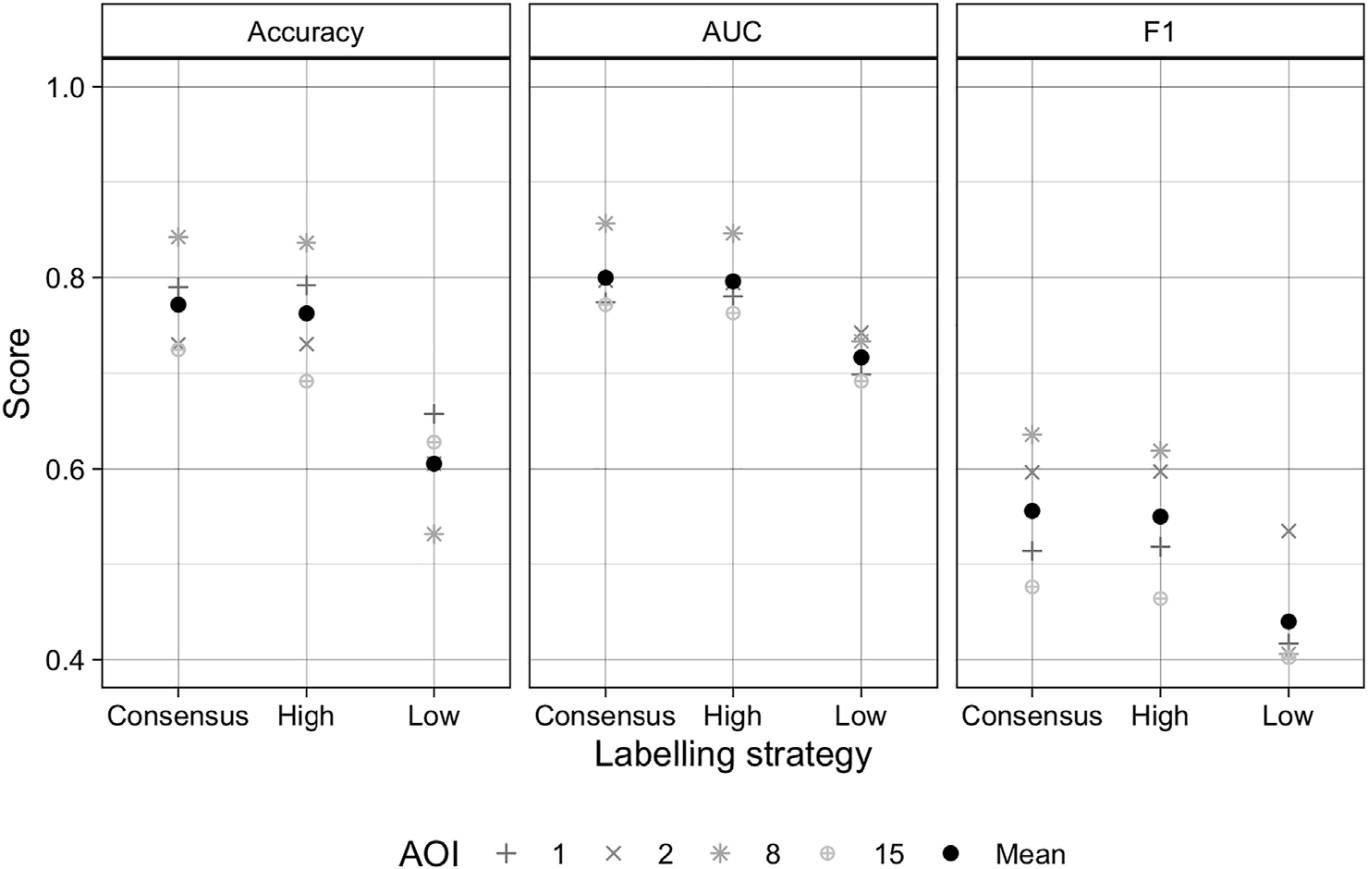
Scores for overall accuracy, area under the curve of the Receiver Operating Characteristic, and the F1 score resulting from models trained with consensus labels, and labels made by the most and least accurate labellers to map each site. Comparisons were made for AOIs 1, 2, 8, and 15, denoted by grey symbols, while the mean scores across these AOIs are shown for each metric.

Treating each labeller’s average label quality scores (Supplementary Figure S10) as a proxy for error, we used these scores to develop training sets to test the impact of label error on model performance. The results of these tests, which were conducted in AOIs 1, 2, 8, and 15, showed that the average accuracy, AUC, and F1 scores for models trained with the consensus labels were respectively 0.772, 0.8, and 0.555 (Figure 5). Performance metrics from consensus-trained models were just 0.5–1.2 percent higher than those models trained with the most accurate individuals’ labels (accuracy = 0.762; AUC = 0.796; F1 = 0.55), but were 11.6–27.4 higher than models trained with the least accurate individual labels (accuracy = 0.606; AUC = 0.716; F1 = 0.44).

Correlations (Supplementary Table S3) between the mean label risk per AOI (Supplementary Figures S11, S12) and model performance metrics showed strong (Spearman’s Rank Correlation = −0.824) to moderate (*r* = −0.568) negative correlations between label risk and accuracy and AUC, respectively, while F1 had a weaker but moderate positive association (*r* = 0.456). The positive sign of the latter relationship is counter-intuitive, but is explained by risk’s association with precision, one of two inputs to F1, which was moderately positive (*r* = 0.629), whereas risk had a negligible correlation with recall (*r* = 0.206), F1’s other component. The correlation between risk and the false positive rate (*r* = 0.688), another important performance metric, shows that labelling uncertainty may increase model commission error.

### 3.3 Map Accuracy

#### 3.3.1 Categorical Accuracy

We used the map reference sample to evaluate the accuracy of the cropland probability map (after classifying it using a threshold probability of 0.5) and the map of segmented field boundary maps. We found that the overall accuracy of the pixel-wise classifications was 88% against this map reference sample (Table 2). Confining the map reference sample to four distinct zones (Supplementary Figure S5A) shows that overall accuracy ranged from 83.3% in Zone 1 (AOIs 1–3) to 93.6% in Zone 3 (AOIs 10, 11, 13, 15, and 16). The Producer’s accuracy of the cropland class was 61.7% across Ghana, ranging from 45.6% in Zone 3–67.9% in Zone 1, while the User’s accuracy was 67.3% overall, ranging from 59.8% in Zone 4–71.4% in Zone 3. Both measures of accuracy were substantially higher for the non-cropland class across all zones, typically exceeding 90%. The lowest accuracies for the non-cropland class was in Zone 1 (Producer’s = 89.3%; User’s = 87.7%).

**TABLE 2:** Map accuracies and adjusted area estimates for the 3 m pixel-wise classifications (based on Random Forests predictions; top 5 rows) and the segmented map (bottom 5 rows). Results are provided for 4 zones (Zone 1 = AOIs 1–3; Zone 2 = AOIs 4–9; Zone 3 = AOIs 10, 11, 13, 14, 16; Zone 4 = AOIs 12, 15) plus the entire country. The error matrix (with reference values in columns) provides the areal percentage for each cell, and the Producer’s (P), User’s (U), and overall (O) map accuracies and their margins of error (in parenthesis) are provided, as well as the sample-adjusted area estimates (in km^2^) and margins of error.

			Non-crop	Crop	Total	U	O	n	Area
Per-pixel classification		Non-crop	64.2	9	73.2	87.7 (5.5)	83.3 (4.3)	138	40,992 (2,468)
		Crop	7.7	19.1	26.8	71.2 (5.9)		226	16,025 (2,468)
	Zone 1	P	89.3 (5.5)	67.9 (5.9)					
		n	186	178					
		Non-crop	73.9	6.7	80.6	91.7 (4.2)	86.5 (3.6)	169	65,123 (2,866)
		Crop	6.8	12.6	19.4	64.8 (6.0)		247	15,533 (2,866)
	Zone 2	P	91.5 (4.2)	65.3 (6.0)					
		n	242	174					
		Non-crop	89.6	4.8	94.4	94.9 (3.2)	93.6 (3.1)	177	70,885 (2,413)
		Crop	1.6	4	5.6	71.4 (9.0)		98	6,860 (2,413)
	Zone 3	P	98.2 (3.2)	45.6 (9.0)					
		n	196	79					
		Non-crop	80.7	5.3	85.9	93.8 (5.9)	89.1 (5.3)	65	26,473 (1,615)
		Crop	5.7	8.4	14.1	59.8 (10.4)		87	4,199 (1,615)
	Zone 4	P	93.4 (5.9)	61.4 (10.4)					
		n	96	56					
		Non-crop	77.2	6.7	83.9	92.0 (2.3)	88.0 (2.0)	549	202,856 (4,904)
		Crop	5.3	10.8	16.1	67.3 (3.6)		658	43,233 (4,904)
	Ghana	P	93.6 (2.3)	61.7 (3.6)					
		n	720	487					
		Non-crop	57.6	4.2	61.8	93.2 (5.3)	81.4 (3.9)	88	40,890 (2,236)
		Crop	14.4	23.8	38.2	62.3 (5.7)		276	15,905 (2,236)
Segmentation	Zone 1	P	80.0 (5.3)	84.9 (5.7)					
		n	186	178					
		Non-crop	70.4	3.7	74.1	95.0 (3.9)	85.2 (3.2)	121	65,642 (2,599)
		Crop	11.2	14.8	25.9	56.9 (5.7)		295	14,841 (2,599)
	Zone 2	P	(3.9)	80.1 (5.7)					
		n	242	174					
		Non-crop	86.6	3	89.6	96.6 (2.9)	92.6 (2.8)	148	71,695 (2,181)
		Crop	4.3	6.1	10.4	58.3 (8.6)		127	7,167 (2,181)
	Zone 3	P	95.2 (2.9)	66.7 (8.6)					
		n	196	79					
		Non-crop	75.3	3.4	78.7	95.7 (6.0)	86.1 (5.1)	46	26,712 (1,593)
		Crop	10.4	10.8	21.3	50.9 (9.6)		106	4,446 (1,593)
	Zone 4	P	87.8 (6.0)	76.0 (9.6)					
		n	96	56					
		Non-crop	73.2	3.6	76.8	95.3 (2.1)	86.7 (1.8)	403	204,940 (4,395)
		Crop	9.7	13.5	23.2	58.2 (3.4)		804	42,359 (4,395)
	Ghana	P	88.3 (2.1)	78.9 (3.4)					
		n	720	487					

The overall accuracies obtained from the segmented maps were generally 1-2 percentage points lower than those of the per-pixel maps, while User’s accuracies tended to be 8–10 percentage points less (Table 2). In contrast, Producer’s accuracies were 15–20 points higher than in the per-pixel map. The segmentation step therefore helped to reduce omission error while substantially increasing commission error.

#### 3.3.2 Segmentation Quality

The comparisons of digitized versus segmented field boundaries showed that the mean field size across all validation sites averaged 4.97 ha (Median = 3.75; StDev = 6.04), which was 1.41 times larger than the 2.06 ha (Median = 1.35; StDev = 3.26) mean area of labeller-digitized polygons. This discrepancy was primarily caused by results in four AOIs (2, 3, 7, and 15; Supplementary Figure S14), where segments averaged between 7.76 and 10.76 ha, compared to 2.18–2.77 ha for the corresponding hand-digitized polygons. The number of segmented fields per validation site averaged 3.08 (median = 2.66; StDev = 2.9) compared to 4.4 (median = 3.38; StDev = 4.52) for digitized polygons (Supplementary Figure S15).

### 3.4 Ghana’s Croplands

**FIGURE 6:**
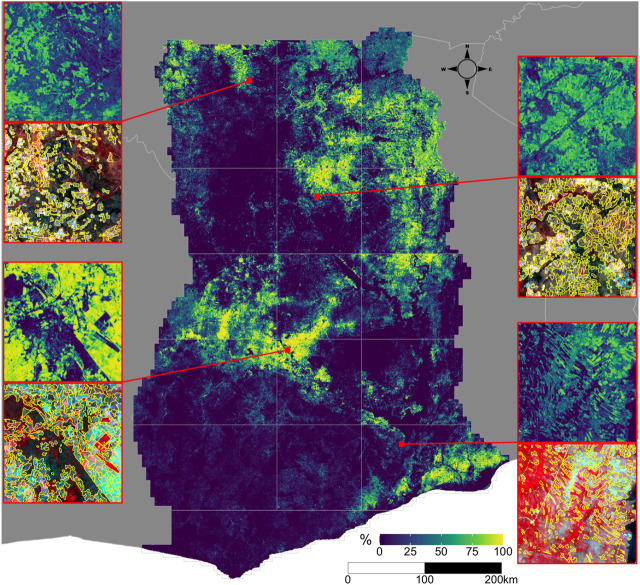
The distribution of croplands in Ghana. The main map shows the percentage of croplands in each 0.005° grid cell, derived from the predicted cropland probabilities. The insets on the margins illustrate predicted probabilities (top map in each couplet) at original image resolution (0.000025°) and segmented field boundaries overlaid on the dry season PlanetScope composite, for four separate tiles. Each tile’s position is shown on the main map by the red line.

Two separate maps of cropland were produced for each AOI, a per-pixel map derived from the cropland probabilities, and the vectorized map of field boundaries (Figure 6). The former provides the more accurate picture of cropland distributions in Ghana, which are most concentrated in the Southeastern corner (AOI 15), the central-western region (AOI 7, the northeastern and northwestern corners of AOIs 10 and 11, and the south of AOI 8), and the northeastern quadrant stretching from AOI 9 through AOIs 5 and 6 and up to AOIs 2 and 3. The northern third of AOI 1 also has noticeable densities of cropland. Several prominent areas of low cropland density indicate the presence of large protected areas, such as Mole National Park in the southeastern corner of AOI 1 and Digya National Park in the northwestern corner of AOI 12. The relative absence of cropland in AOIs 13, 14, and 16 does not reflect the scarcity of agriculture in these areas, but rather the predominance of tree crops, which we did not map.

Using the map reference sample and each map, we made two separate estimates of the total cropland area in Ghana in 2018. The cropland extent estimated from the field boundary map was 42,359 km^2^ (with a margin of error of 4,395 km^2^), or 17.1% (15.4–18.9%) of the mapped area. The estimate from the per pixel map was 43,233 km^2^ (margin of error = 4,904 km^2^), or 17.6% (15.6–19.6%) of area.

The field boundary map provides additional information on how the characteristics of croplands vary across Ghana, ranging from narrow, strip-like fields in parts of AOI 15 (Figure 6’s lower right inset) to more densely packed, less distinctly shaped fields in AOI 5 (upper right inset in Figure 6). To explore how field characteristics varied geographically, we mapped the average size and total number of fields within each 0.05° tile grid (Supplementary Figure S16). These patterns generally correspond to those seen in the cropland density map (Figure 6), with larger sizes and field counts occurring where field densities were higher, although the biases (relative to the validation labels) in both measures (Supplementary Figures S14, S15) complicate interpretations of those variations. To minimize this complication, we used the calculated biases to develop adjusted estimates of field size and count (Table 3). These adjusted estimates show that the typical field size in Ghana is 1.73 ha, ranging from 0.96 in AOI 13 to 2.82 ha in AOI 4, with fields in the forest zone AOIs (10, 11, 13, 14, 16) generally smaller than those in the northern half of the country (Table 3). The estimated total number of fields is 1,662,281, or 205 fields per tile grid cells, varying from 108 fields/tile cell in AOI 4 to 399 in AOI 6.

**TABLE 3:** The average size and total number of crop fields for each AOI and for Ghana overall. The original and bias-adjusted values for each measure are provided, as well as the total number of 0.05° degree tiles in each AOI.

AOI	N tiles	Size	Size (adj)	N	N/tile	N (adj)	N (adj)/tile
1	777	3.71	1.26	97,822	126	127,580	164
2	597	7.66	1.96	87,666	147	120,651	202
3	501	8.24	2.18	108,819	217	104,422	208
4	465	2.44	2.82	26,276	57	50,163	108
5	400	4.24	2.09	43,290	108	53,756	134
6	429	5.10	2.15	81,363	190	145,347	339
7	471	5.64	1.49	93,282	198	123,005	261
8	400	4.89	1.98	55,500	139	78,868	197
9	479	4.10	1.82	72,081	150	89,840	188
10	630	2.24	1.04	119,019	189	170,907	271
11	400	3.65	1.52	52,510	131	94,709	237
12	471	3.44	1.77	44,667	95	52,947	112
13	627	0.84	0.96	67,996	108	125,368	200
14	400	1.09	2.72	56,006	140	101,767	254
15	548	4.95	1.54	75,752	138	105,681	193
16	521	0.95	1.41	49,097	94	117,268	225
Ghana	8,116	3.92	1.73	1,131,146	139	1,662,281	205

## 4 Discussion

These results demonstrate a capability to map the characteristics of smallholder-dominated cropping systems at high spatial resolution, annual time steps, and national scales. The resulting maps provide an updated and more granular view of the distribution and extent of croplands in Ghana, complementing existing national to regional land cover maps derived from moderate resolution imagery (Hackman et al., 2017; Xiong et al., 2017; ESA nd.). Those prior studies estimated that cropland covers 19.4 (Xiong et al., 2017) to 32% (Hackman et al., 2017) of Ghana in 2015. In contrast, our 2018 maps provide a raw estimate of 16.1–23.2% cover (Table 2), and our map reference sample-based estimate was 17.1–17.6%. Our results thus suggest that Ghana’s croplands are less extensive than those previous estimates. However, this difference may arise from our use of a cropland definition that excludes longer fallows and abandoned fields, which in some regions may comprise over half of total cropland area (Tong et al., 2020).

In addition to this updated information on Ghana’s cropland extent and distribution, our results provide new insights into field size and number at a national scale (Figure 6, Supplementary Figures S14, S15). Previous efforts to map smallholder field boundaries have either used *in situ* data collection (Carletto et al., 2013, Carletto et al., 2015) or remote sensing studies over relatively small (e.g. Forkuor et al., 2014; Persello et al., 2019) or discontiguous (Estes et al., 2016a) areas. The most extensive studies to date enlisted crowdsourced volunteers to classify fields visible within high resolution imagery sampled from virtual globes into broad size categories (Fritz et al., 2015; Lesiv et al., 2019). Those efforts included country-specific results for Ghana (*n* = 263), which yield an average field size estimate of 5.33 ha^9^. This estimate exceeds our Ghana-wide average segment size (3.92 ha; Table 3), but is closer to the mean (4.97 ha) within AOIs 1–9, 12, and 15, which is where most of the crowdsourced sample appears to have been collected. However, our bias-adjusted estimates of 1.73 (Ghana-wide) and 1.87 (AOIs 1–9, 12, and 15) ha were much smaller.

### 4.1 Map Accuracy and Key Sources of Error

Although these maps provide valuable new information, they nevertheless contain substantial errors that can impact “downstream” uses (e.g., estimating crop production estimates) and decisions based on these maps in unpredictable ways (Estes et al., 2018). The overall accuracies (86.7–88%, Table 2) are near the boundary of what might be considered achievable map accuracy (Elmes et al., 2020), given that we only have ∼85% confidence in our map reference sample, which is our best estimate of the “truth.” However, accuracies for the cropland class were much lower, falling between 62 (producer’s) to 67 (user’s) percent country-wide for the per-pixel map (Table 2), meaning the model produced substantial commission and omission errors for this class. The segmented boundary maps had fewer omission errors (producer’s accuracy = 79%), but higher false positives (user’s accuracy = 58.2%). These accuracies are near the middle to upper ranges of those reported for the cropland class in other large-area mapping studies (Hackman et al., 2017; Lesiv et al., 2017; Xiong et al., 2017).

The patterns of accuracies within the cropland class varied by zone. These zones largely align, albeit with some discrepancies, with the country’s agro-ecological zones (AEZs), thus the accuracy patterns may be in part because some regions are simply more difficult to map. Producer’s accuracy for both maps was highest in the two northern zones (1 and 2), which are primarily savannas (Supplementary Figure S5), and lowest in zones 3 and 4, which are comprised of forest or coastal savannas. User’s accuracy followed a similar pattern, with the exception of Zone 3, which had the highest user’s accuracy, albeit from a very small sample. Aligning the reference samples more precisely with agroecozone boundaries (Supplementary Figure S5B) provides further insight into error patterns within the per-pixel map’s cropland class (Supplementary Table S4). Coastal savannas in the southeast had the highest producer’s and lowest user’s accuracy, perhaps because this region has high density cropland inter-mixed with uncultivated areas that have low woody cover, which could help promote commission error. Maps in the northern savannas had the best balance between omission and commission error, and had the highest overall user’s accuracy. The transitional zone between forest and savanna had a very low Producer’s accuracy (21%), which likely reflects the fact that it was divided between several AOIs for mapping (Supplementary Figure S5), and thus was under-represented in the training samples, particularly in AOIs 10 and 11 (Supplementary Figure S7B).

Beyond the errors linked to regional differences, several other important factors contributed to map error. The first of these related to the mapping extent and image resolution. Given the goal of developing a high resolution, country-scale map, the large data volume constrained us to use a relatively small feature set and less than the recommended tree number and depth (Maxwell et al., 2018) in our Random Forests models, in order to limit computational costs. Previous work found that Random Forests achieves much better performance on small-scale croplands when trained on a much larger number of features (Debats et al., 2016; Lebourgeois et al., 2017). However, applying such a large feature set within the extent of our AOIs was not possible, as the computing time and costs would have been several times larger^10^. This reduced the skill of the model, particularly when it came to differentiating cropland from bare or sparsely vegetated patches, which were common in many AOIs.

The inherent difficulty of the labelling task was another major limiting factor. Our platform was designed to minimize label errors, but determining croplands from non-croplands in these agricultural systems can be difficult. Labellers had to evaluate multiple image sources and to rely heavily on their judgment, which inevitably led to errors. Interpretation was particularly hard where croplands and surrounding landscapes had similar dry season reflectances, which was a particular problem in the northernmost savannas. Smaller field sizes also complicated labelling, as these become increasingly indistinct in the ∼4 m PlanetScope composites. The difficulty of labelling is reflected in the magnitude of the Bayesian Risk metrics (Supplementary Figures S11, S12), and by the average assignment quality scores of each labeller (71%; Supplementary Figure S10). Although prior work (Rodriguez-Galiano et al., 2012; Mellor et al., 2015) found that Random Forests are robust to label error, we found that it has substantial impact (Figure 5), which suggests that improving label quality is one of the most important factors in increasing model accuracy. Newer models, such as convolutional neural networks, may be less sensitive to label error, provided the error is random and the map reference samples are of high quality (Burke et al., 2021). However, over many smallholder systems training label errors will likely be biased in a particular direction (e.g. towards omission when fields are not easily distinguished from the background), and our results show that reference labels can have substantial uncertainty.

Image quality was another issue, although primarily in the forested AOIs, where frequent cloud cover and the corresponding lower number of available images resulted in lower quality composites (Figure 3), with more brightness artifacts and blur. This impacted labeller’s abilities to discern fields, and doubtless affected model predictions. Little can be done to mitigate these errors, short of confining imagery to the less cloudy dry season, which could reduce model performance by removing the temporal contrast (Debats et al., 2016; Defourny et al., 2019), or by adding radar data (e.g., Sentinel-1) to the predictor set, which would reduce map resolution. Composite quality could be improved by using imagery from the same seasons over multiple years, but this would undermine the goal of developing annual maps, while the dynamism of the croplands would blur field boundaries within the imagery.

The final major source of error arose from the segmentation process. The vectorized maps had high commission errors caused by uncertainties in the Random Forests predictions. Model uncertainty meant that many pixels in non-cropland areas had probabilities with values near 0.5. Segments in these areas were retained if the average probability of intersected pixels exceeded the 0.5 classification threshold. A more accurate classifier would reduce such errors, as would a locally varying classification threshold (e.g., Waldner and Diakogiannis, 2020). Over-merging was another source of error in the segmentation algorithm, which led to overestimated field sizes and unrealistic shapes in some areas, particularly in high density croplands (e.g. in AOIs 2 and 8; Figure 6) where boundaries between adjacent fields were indistinct in the imagery. Preventing merging could help in such cases, but potentially lead to over-segmentation, thereby underestimating field sizes.

### 4.2 Error Mitigation Features

Despite these numerous sources of errors, our approach was effective in mitigating several of these error sources. Label quality assessment and consensus labelling were the most effective error mitigation tools. Label quality scores allowed us to quantify the impact of label error on model performance (Figure 5), while consensus labels produced maps that were more accurate than they would have been if we had relied on individually generated labels. The quality scores also helped to improve the overall accuracy of consensus labels, by placing higher weight on the work of more accurate labellers. In addition to these benefits, label quality scores (Supplementary Figure S10) also allowed us to select the labels most likely to accurately capture field sizes and numbers, which we used to estimated and correct the biases in these two measures derived from the segmented field boundaries.

Active learning improved overall model performance relative to randomized training site selection, in line with findings from two recent efforts (Debats et al., 2017; Hamrouni et al., 2021). Although the relative performance gains that we observed were smaller (e.g., Debats et al., 2017 found 29% higher model performance after one iteration, and 8% higher on the final iterations), those comparisons were made by starting with a training sample that was <1/10 the size of ours. Our large starter sample meant that the models were substantially trained before they were exposed to actively selected labels, thereby diluting their impact on performance. Nevertheless, we found higher performance from active learning, most notably in the F1 score (Supplementary Figure S9), a balanced performance metric, which further demonstrates its effectiveness.

The detail, temporal precision, and large extent of our maps was enabled by our use of PlanetScope data, which is currently the only source of sub-5 meter imagery with daily coverage (McCabe et al., 2017). These spatio-temporal characteristics, together with the compositing technique we developed, allowed us to create a complete image catalog for Ghana covering the two major seasons in the 2018 agricultural year. Daily revisits were key to this capability, as they increased the number of cloud-free observations that could be collected in each season. Over rainy tropical regions, such as southern Ghana, the odds of obtaining a single clear PlanetScope observation within a 1–2 week period is often less than 50% (Roy et al., 2021). Although Sentinel-2 is free and has sufficient spatial resolution to effectively classify small-scale croplands (e.g. Defourny et al., 2019; Kerner et al., 2020), its 5-days interval is likely too infrequent to generate adequate seasonal composites over much of Ghana. The smaller number of clear observations that Sentinel-2 can provide compared to a daily acquisition schedule would result in greater reflectance discontinuities and residual cloud cover in the resulting composites. Given Ghana’s persistent cloudiness, such artifacts were present in a number of our PlanetScope composites. However, these were not large enough to have an appreciable impact on the resulting maps, as the mapping algorithms appeared to be relatively insensitive to these anomalies and any discontinuities along tile boundaries.

### 4.3 Lingering Questions

Several potential issues not addressed in our assessment merit further exploration. One of these was the degree of correspondence between image- and ground-collected labels. However, such comparisons may reveal unresolvable discrepancies between the two perspectives. The highly dynamic nature of these agricultural systems means that relatively narrow differences between the dates of ground- and image-based label collection can lead to substantial disagreement, simply because the fields themselves may have shifted during the interval (Elmes et al., 2020). These discrepancies can be exacerbated by the definition used to determine what constitutes a field, which might vary on the ground depending on who is being asked, or who is doing the collecting. These factors suggest that difference between ground- and image-collected labels would not necessarily indicate how far image labellers were from the “truth.” Nevertheless, a comparison against ground data would help to assess how accurately image-collected labels capture the typical size of fields, and thus merits further investigation.

The temporal discrepancies mentioned above (and discussed in Elmes et al., 2020) are another reason why we chose not to label on basemap imagery (in addition to restrictive usage terms), which is typically several years old (Lesiv et al., 2018). However, we did not assess whether the higher label accuracy one might achieve by digitizing on a <1–2 m resolution basemap would offset model errors caused by temporal mismatches.

Another potential issue is the degree to which our assessment of label error on model performance (Figure 5) was influenced by the validation dataset we used, which was based on consensus labels. This could have confounded the analysis, particularly when comparing the consensus label-trained models with those trained with the most accurate individual labels. However, a visual assessment of the resulting probability maps confirms that models trained with the consensus and most accurate individual labels were more precise than the model trained with lower quality labels (Supplementary Figure S13).

## 5 Conclusion

This work demonstrates a proof of concept for developing high resolution, annual maps of field boundaries in smallholder-dominated croplands at national to regional scales. The approach we used to develop these maps includes a novel procedure for compositing daily PlanetScope imagery, and follows recommended best practices for training and assessing machine learning models (Elmes et al., 2020). Using PlanetScope data enabled us to make a country-wide, high-resolution, two-season catalog of imagery for a single year, even over areas with frequent cloud cover. Methods to assess labeller accuracy and to create consensus labels helped to minimize training label errors, which in turn substantially improved the accuracy of the mapping model. Model performance was further enhanced by using an active learning framework, which selects the most informative training sample. This mapping approach can be readily updated to integrate improvements, such as newer machine learning models.

The resulting maps provide valuable information on Ghana’s agricultural systems, including an updated estimate of cropped area, and new data on the characteristics of Ghana’s cropping systems, such as the sizes and numbers of fields. Beyond the information about field characteristics, field boundary maps can help improve remote estimation of crop areas and yield (e.g. Estes et al., 2013), and provide deeper insights into important socioeconomic aspects of agricultural systems, such as the relationships between agricultural productivity and farm size (Feder, 1985; Carletto et al., 2013; Desiere and Jolliffe, 2018). Such maps will be important for understanding the rapid agricultural change that is currently occurring in Africa.

## Data Availability

The maps presented here are a version 1 product that is freely available to use, along with its underlying code. The names and links of the data and code repositories can be found in the Supplementary Material. In their current form, the maps may be useful for a variety of research applications. For example, analyzing the distributions of values in the probability maps may provide additional insight into the relative extents of active versus fallow croplands (Tong et al., 2020). However, use of these data for inventory estimates, to develop other map products, or to guide decision-making should be made with caution and account for the reported errors (Olofsson et al., 2014; Estes et al., 2018; Stehman and Foody 2019).

## Footnotes

1
www.planet.com/markets/education-and-research/

2 assets.planet.com/docs/Planet_ParticipantLicenseAgreement_NICFI.pdf

3
https://wiki.openstreetmap.org/wiki/Slippy_Map

4
https://docs.aws.amazon.com/emr/latest/APIReference/emr-api.pdf

5
https://github.com/locationtech/geotrellis

6
https://rasterio.readthedocs.io/en/latest/

7
https://rasterframes.io/

8
https://www.cogeo.org/

9 Obtained by calculating the weighted mean from the count of the five size classes and the mean of the hectare range provided for the four smallest size classes, and the lower bound of the size range provided for the largest size class. Data sourced from Supplementary Table S3 in Lesiv et al., 2019.

10 Each active learning iteration ran for ∼4–8 h on 800 CPUs, followed by a final ∼10–14 h for prediction
