# Supplementary material for: High Resolution, Annual Maps of Field Boundaries for Smallholder-Dominated Croplands at National Scales
Source: Front Artif Intell. 2022 Feb 25;4:744863. doi: 10.3389/frai.2021.744863 (PMC8916109; doi:10.3389/frai.2021.744863)
Supplement: Supplementary file 1 [file DataSheet1.pdf]

High resolution, annual maps of field boundaries for smallholder-dominated croplands at national scales

Supporting Information

Contents

1 Code and Data Availability **2**

2 Supplemental Methods **2**
2.1 Map reference system . . . 2
2.2 Image compositing . . . 2
2.2.1 Variation in lengths of temporal window used . . . 2
2.2.2 Assessing composite quality . . . 2
2.3 Mapping platform . . . 3
2.3.1 Digitizing tools . . . 3
2.3.2 Mapping rules . . . 3
2.3.2.1 Interpreting and mapping imagery . . . 3
2.3.2.2 Choosing what to map . . . 4
2.3.3 Assessing label accuracy . . . 4
2.3.4 Consensus labelling . . . 5
2.3.4.1 Example . . . 7
2.3.5 Segmentation . . . 8
2.3.6 Accuracy assessment . . . 8
2.3.6.1 Map reference sample design . . . 8
2.3.6.2 Response design . . . 9
2.3.6.3 Map reference label uncertainty . . . 10
2.3.6.4 Accuracy assessment zones . . . 11

3 Supplemental Results **11**
3.1 Image catalog and quality . . . 11
3.2 Mapping cropland probabilities with active learning . . . 11
3.2.1 Labelling . . . 12
3.2.2 Model performance . . . 12
3.2.3 The impact of training data error . . . 14
3.3 Map accuracy . . . 15
3.3.1 Categorical accuracy . . . 15
3.3.2 Field area and number . . . 15
3.4 Cropland characteristics . . . 18
3.4.1 Field area and number . . . 18

4 References **22**

1 Code and Data Availability

The repositories containing the data, code, and manuscript source files used to produce this paper are listed below:

- `activemapper`: Repository[1] containing code and derived data used to write this paper.
- `imager`: Repository[2] containing code for the image processing software.
- `labeller`: Repository[3] containing code for the labelling platform.
- `learner`: Repository[4] containing code for the active learning component.
- `segmenter`: Repository[5] containing code for the segmentation algorithm.
- `mappingafrica.io`: Project website[6] linking to web map displaying field boundary maps. The web map is currently undergoing redevelopment, and will be hosted on a new URL and links download maps. In the interim, map data are available on request.

2 Supplemental Methods

2.1 Map reference system

The map reference system used in our mapping approach had three different levels. At the coarsest level, Ghana was divided into 16 different mapping zones, or areas of interest (AOIS; Figure S1A), that were used to create AOI-specific mapping models. Each AOIs was comprised of 400 to 777 adjacent tiles. These tiles were used to create seasonal image composites, and were defined within a 0.05 degree grid (Figure S1B)), with each tile numbered to correspond to a larger 1 degree grid cell that it sits within (dotted lines in Figure S1B). Training and reference labels were created within a 0.005 degree grid that nested within each tile (Figure S1C). Therefore, each tile has 100 grid cells, and there are 400 tiles per 1X1 degree. The smallest AOI consists of a single 1X1 degree, which fall in the center of the country (AOIs 5, 8, 11, and 14). AOIs falling along Ghana's boundaries were created by tiles from 1X1 degree cells that straddled Ghana's border with those from the closest degree cells that were fully contained within Ghana (e.g. AOI 1), with the exception of AOI 16, which was comprised of tiles in three partial 1X1 degree cells along Ghana's coast.

2.2 Image compositing

2.2.1 Variation in lengths of temporal window used

The typical window for compositing dry season imagery was December, 2018 to February, 2019, but in the cloudiest regions (AOIs 10, 11, 13, 14, 16) we started the dry season window in November, to ensure a sufficient density of images for compositing.

2.2.2 Assessing composite quality

The rubric presented in Table S1 was used to assess the quality of the seasonal image composites. The imagery was evaluated by examining their Raster Foundry (Azavea 2020) overlays within a `labeller` instance set up for the purpose.

[1]github.com/agroimpacts/activemapper
[2]github.com/agroimpacts/imager
[3]github.com/agroimpacts/labeller
[4]github.com/agroimpacts/learner
[5]github.com/agroimpacts/segmenter
[6]https://mappingafrica.io

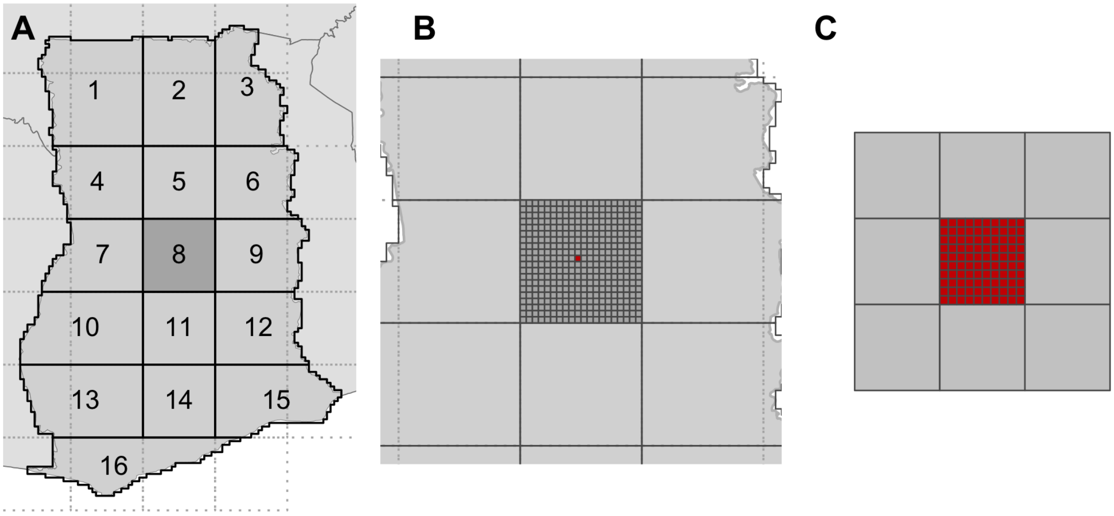

Figure S1: The reference system used in this mapping approach, including A) numbered areas of interest (AOIs) that define the minimum mapping geography (solid black lines; dotted lines indicate boundaries of 1 degree grid), B) the 0.05 degree tile used for compositing imagery, and C) the 0.005 degree resolution reference grid used for collecting training data and distributed computing.

Table S1: Four dimensions used to assess the assess the quality of the temporally composited image tiles, including the criteria used to award points for scoring each dimension.

Quality dimension	3 pts	2 pts	1 pts	0 pts
Percent affected by residual cloud	<1%	1-5%	5-10%	>10%
Percent affected by cloud shadow	<1%	1-5%	5-10%	>10%
Number of visible scene boundaries	None	1	2-3	>3
Percent blurred	None	<20%	20-50%	>50%

2.3 Mapping platform

2.3.1 Digitizing tools

To minimize the risk of topological errors, *mapper*'s polygon digitizing tools prevent drawing that results in self-intersections and overlaps between adjacent polygons. Upon submission, the PostGIS `ST_MakeValid` function is applied to each polygon's geometry to clean remaining topological errors upon insertion into the database.

2.3.2 Mapping rules

Labellers followed set of rules described in the next two sections when interpreting and digitizing field boundaries.

2.3.2.1 Interpreting and mapping imagery

- Map the fields that are visible in the PlanetScope images, not the fields that you see in the basemap.
- As the first choice, map the field outlines as they appear in the dry season PlanetScope images. If a field is not clearly visible in the dry season image, but is visible in the growing season image, then the next choice is to map it in the growing season image.

- Use all available images (PlanetScope scenes and base map) to help you interpret what is and is not a field. Toggling back and forth between the different image sources can help identify and classify the field boundaries.
- Map fields that intersect the mapping target, making sure to finish the entire boundary.

2.3.2.2 Choosing what to map

- Draw polygons around fields that look like they contain crops that are planted and harvested during a single year (or occasionally slightly longer, in the case of crops such as sugarcane). These fields may look recently ploughed or harvested, or contain actively growing crops.
- Do not draw polygons around fields that are under tree crops, such as orchards, woodlots, or other planted forest types.
- Do not draw polygons around fields that look like they are annual croplands, but are overgrown and haven't been planted for a few years. These are possibly fallows or abandoned fields.
- Do not draw polygons if you cannot tell whether a piece of land is an annual crop field or if it is perhaps another type of land cover (e.g. bare land, or a young orchard).
- Do not draw any polygons if there are no annual crop fields to map.

2.3.3 Assessing label accuracy

For each accuracy assessment assignment, the labeller's maps are scored against training reference polygons digitized by one of the map supervisors (e.g. Estes or Ye). A proportion of training reference sites were of the non-cropland class and thus had no corresponding polygons.

As described in the main text, the score for a particular assignment i is calculated as the weighted sum of five accuracy metrics:

$$score_i = \beta_0 \mathrm{I} + \beta_1 \mathrm{O} + \beta_2 \mathrm{F} + \beta_3 \mathrm{E} + \beta_4 \mathrm{C}$$

Where β_{0-4} are the weights assigned to each accuracy metric that sum to 1. For the current production version, β_{0-4} were assigned as 0.4, 0.2, 0.2, 0.1, and 0.1.

I is "inside the box" accuracy which is defined as a proportion of the area correctly mapped within a 0.005 degree resolution grid (I_c) over total "inside the box" area for this grid (I_t).

$$I = \frac{I_c}{I_t}$$

O is "outside the box" accuracy which refers to a proportion of the field area correctly mapped outside the grid over total "outside the box" region (O_t, the region within the bounding box of the workers' polygons but not within 0.005 degree resolution grid).

$$O = \frac{O_c}{O_t}$$

F is the fragmentation accuracy which is defined as a proportion of matched polygon number (N_m, the number of the workers' polygons that has at least 50% of its region overlapped with a reference polygon) over total workers' polygon number (N_t).

$$F = \frac{N_m}{N_t}$$

E is the average edge accuracy for all pairs of matched workers and reference polygons; the edge accuracy for a single pair is defined as the length of 'correctly mapped edges' (L_c, the partial boundary of a workers' polygon that are within a three-pixel buffer region of the matched reference polygon boundary) over the total boundary length of its matched reference polygon (L_t).

$$E = \frac{L_c}{L_t}$$

C is the categorical accuracy, i.e., a proportion of the area that has been correctly labeled with field category within intersected regions between worker's and reference polygons (T_c) over the total intersecting area (T_t).

$$C = \frac{T_c}{T_t}$$

2.3.4 Consensus labelling

As described in the main text, the formula used for creating a consensus label is:

$$\mathrm{P}(\theta|\mathrm{D}) = \sum_{\mathrm{i=1}}^{\mathrm{n}} \mathrm{P}(\mathrm{W_i}|\mathrm{D})\mathrm{P}(\theta|\mathrm{D}, \mathrm{W_i}) \tag{1}$$

Where θ represents the true cover type of a pixel (field or not field), D is the worker's label of that field, and W_i is an individual worker. Looking in greater details at this equation, the first half of the righthand side of the equation, $\mathrm{P}(\mathrm{W}_i|\mathrm{D})$, is the "prior" for worker i for the current site based on their history of scores from accuracy assessment assignments. The second term, $\mathrm{P}(\theta|\mathrm{D}, \mathrm{W}_i)$, is the probability that the actual class of the pixel in the current assignment is the class that worker i says that it is, which is either 0 or 1. There are four possible values for this second term:

$$P(\theta = field|D_i = field) = 1 \tag{2}$$
$$P(\theta = nofield|D_i = field) = 0 \tag{3}$$
$$P(\theta = nofield|D_i = nofield) = 1 \tag{4}$$
$$P(\theta = field|D_i = nofield) = 0 \tag{5}$$

Where equations 2 and 4 represent true positives and negatives, respectively, and equation 3 is a false positive, and equation 5 is a false negative.

Coming back to the first term, the calculation of prior probability can be re-expressed as:

$$\mathrm{P}(\mathrm{W_i}|\mathrm{D}) \approx \mathrm{P}(\mathrm{D}|\mathrm{W_i})\mathrm{P}(\mathrm{W_i}) \tag{6}$$

Where:

$$P(D|W_i) \propto \exp\left(-\frac{1}{2}BIC_i\right) \tag{7}$$

With BIC being the Bayesian information criterion:

$$BIC = \ln(n)k - 2\ln(\hat{L}) \tag{8}$$

In which n is the sample size, k is the number of parameters to estimate, and $\hat{L}$ is the maximum likeihood function. In this case, we are only interested in one parameter (the label that maximizes the likelihood function), thus the BIC becomes:

$$BIC \approx -2\ln(\hat{L}) = 2\ln(p(D|\hat{\theta}, W)) \tag{9}$$

After rearranging, we have:

$$P(D|W_i) \propto p(D|\hat{\theta}, W_i)) \tag{10}$$

Which is the worker maximum likelihood, which can be computed as:

$$P(\theta = \text{field}|\hat{\theta}, M_I) = P(D = \text{field}|\theta = \text{field}, M_I) = \frac{1}{m}\left(\sum_j^m \frac{tp_j}{tp_j + fn_j}\right) \tag{11}$$

$$P(\theta = \text{nofield}|\hat{\theta}, M_I) = P(D = \text{nofield}|\theta = \text{nofield}, M_I) = \frac{1}{m}\left(\sum_j^m \frac{tn_j}{tn_j + fp_j}\right) \tag{12}$$

Equations 11 and 12 are Producer's accuracies, thus the maximum worker likelihood is equivalent to the worker's average Producer's accuracy.

The other component of equation 6, $P(W_i)$, is the worker's average score over m accuracy assessment assignments:

$$P(W_i) \propto \frac{1}{m}\sum_{j=1}^{m} \text{score}_j \tag{13}$$

Thus equation 6 uses two measures of worker accuracy, 1) their overall average accuracy score multipled by 2) their average Producer's accuracy to create a *weight* for their individual maps for the given site. Equation 1 becomes:

$$P(\theta|D) = \frac{\sum_{i=1}^{n} \text{weight}_i P(\theta|D, W_i)}{\sum_{i=1}^{n} \text{weight}_i} \tag{14}$$

With $P(\theta|D, W_i)$ being either 0 or 1. In labelling, if the consensus result for a pixel is: $P(\theta = \text{field}|D) > 0.5$, then we assign that pixel to the field category, otherwise to the no field category.

After creating the consensus label, the degree of confidence in the resulting label value is measured by Bayesian Risk:

$$r = C(1 - L) + (1 - C)L \tag{15}$$

Where C is the consensus probability that a given pixel is a field ($P(\theta = \text{field}|D)$), and L is the consensus label (i.e. non-field if $C < 0.5$, field if $C > 0.5$) for that pixel. The risk values across the entire sample site can be processed in two ways to provide useful information about the confidence in the consensus label for that site. The first is a simple average of all risk values in the site, where the slope of the risk varies depending on whether L is a field or not a field (Figure S2). The closer to 0 the lower the risk that the L is mislabelled, while values approaching 1 indicate increasing risk of mislabelling. A second approach is to calculate the proportion of pixels having risk values that exceed a certain threshold.

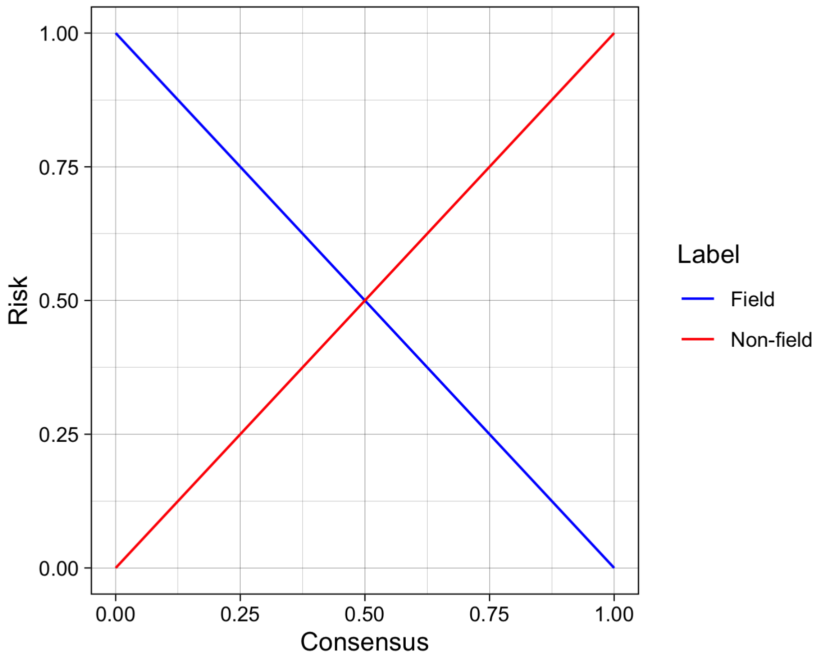

Figure S2: Bayesian risk values (Y-axis) for consensus values (X axis) ranging from 0 to 1 (0 indicates no consensus that a pixel falls into the field class, 1 means complete consensus) for field and non-field consensus labels.

Accuracy assessment and consensus label generation is performed using an `R` package `rmapaccuracy` that is internal to the labelling platform. The package relies on spatial data classes and operations provided by the `sf` (Pebesma 2018) and `raster` (Hijmans 2020) packages.

2.3.4.1 Example To provide an example of this approach in practice, we'll imagine two workers A and B, each with the following histories:

Worker	Prod. Acc. (Field)	Prod. Acc (no field)	Score
A	0.80	0.81	0.75
B	0.62	0.61	0.60

In this scenario, worker A thinks that the given pixel falls within a field, and worker B thinks it is not a field. First, we calculate the weights for each worker:

$$Weight_A = score_A * PA_A(field) = P(W_A)P(D = field|W_A) = 0.8 * 0.75 = 0.6$$

$$Weight_B = score_B * PA_B(field) = P(W_B)P(D = nofield|W_B) = 0.61 * 0.6 = 0.366$$

And then we plug these weights into the full equation:

$$\mathrm{P}(\theta|\mathrm{D}) = \frac{\sum_{\mathrm{i}=1}^{\mathrm{n}} \mathrm{weight_i P(L = field|D, W_i)}}{\sum_{\mathrm{i}=1}^{\mathrm{n}} \mathrm{weight_i}} = \frac{0.6 * 1 + 0.366 * 0}{0.6 + 0.366} = 0.621$$

Since $0.621 > 0.5$, we label the particular pixel a field.

Using equation 15, the corresponding risk associated with this particular pixel's label is thus 0.379 (i.e., 1 - 0.621).

2.3.5 Segmentation

An overview of the two inputs to and outputs from the segmentation algorithm are shown in Figure S3.

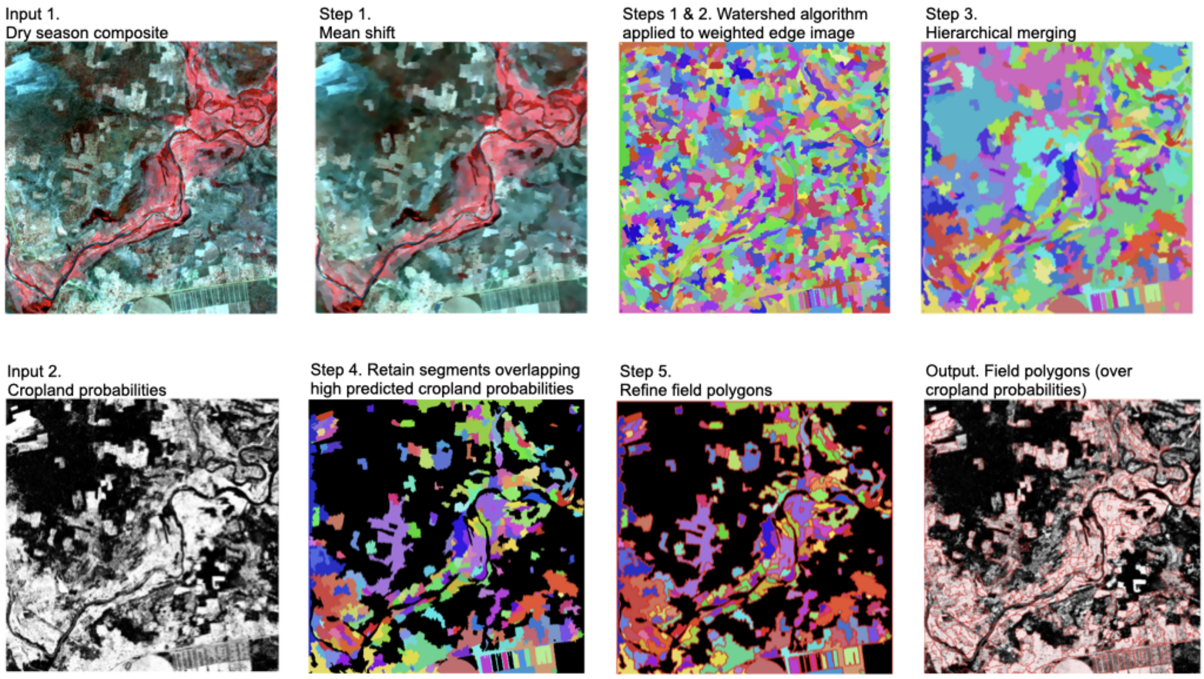

Figure S3: An overview of the inputs and key outputs from the five-step segmentation algorithm.

2.3.6 Accuracy assessment

We designed and implemented a map accuracy assessment protocol following procedures summarized by Stehman and Foody (2019). This entailed the creation of a map reference sample, which first entailed designing a sample, and then designing how the sample response would be collected.

2.3.6.1 Map reference sample design We employed a stratified design for collecting the map reference sample, using the segmented field boundaries to define the strata for cropland/non-cropland. To create the sample, we first extracted the centroids for each of the

sample grid cells in Ghana. We then intersected the centroid points with the field segments, assigned a class of 1 (cropland) where points intersected a field, and 0 where they didn't (non-cropland). We removed from this set of points all those that corresponded to model training, validation, or training reference sites, and extracted a random sample from both the cropland and non-cropland points. To determine the sample size of each, we specified a desired confidence interval using the following formula (Stehman and Foody 2019):

$$n = \frac{z^2 p(1-p)}{d^2}$$

Where p is the estimated probability (or mapped class accuracy), and d is the size of the margin of error (1/2 the confidence interval). We selected a d value of 0.03 and assumed that the user's accuracy of the field class would be 0.75 and that of the non-cropland class would be 0.8, returning sample sizes of 800 and 683, respectively. The distribution of the resulting map reference sample is shown in Figure S4.

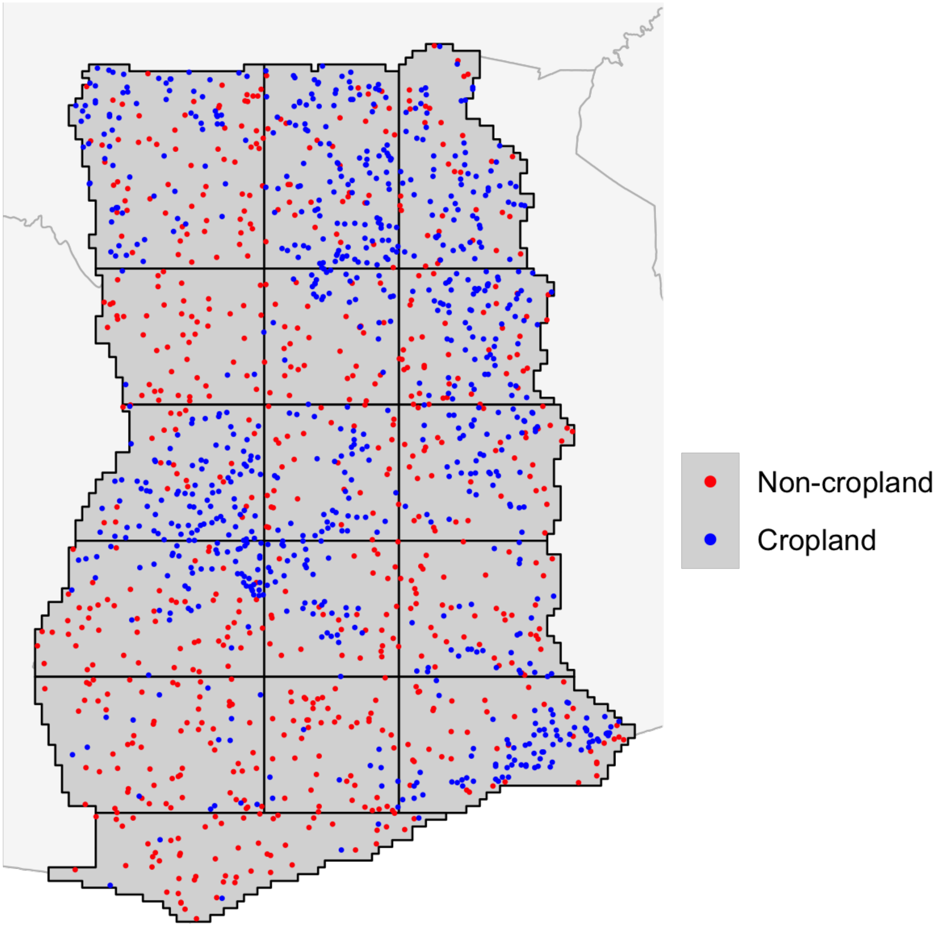

Figure S4: Distribution of the selected map reference sample for the cropland and non-cropland class. Classes represent the values extracted from the map strata, rather than those assigned during the classification of the sample.

2.3.6.2 Response design We collected the map reference sample on a separate instance of the labelling platform set up for the purpose. For the sampling unit, we selected a rectangular polygon of ~0.1 ha (0.0002866° resolution). This polygon was centered on the centroid of each grid cell selected for map reference sample, and was presented as the target grid in the labelling

platform. Four classes were established for the validation: **cropland**, **non-cropland**, **uncertain but likely cropland**, and **uncertain but likely non-cropland**. The latter two classes were designed to capture information related to swidden dynamics, following the rationale that uncertainty and time since last cropping are likely to be positively correlated. This uncertainty also captures information about the inherent difficulty of the mapping task. Samples were collected by visually interpreting the overlays of PlanetScope composites, following the same interpretation protocols used by the labelling team, with the exception that the polygons placed were square and of 0.0002866° resolution.

Two supervisors (Ye and Estes), who were not involved in collecting training and validation labels, collected the map reference sample by evaluating the PlanetScope composites at each site to determine its class membership. The following set of rules were followed in collecting the sample:

1. When determining the class corresponding with the initial location of the target grid, if:

- More than half of the target falls within what appears to be a clear arable crop field, then classify it as **cropland**;
- More than half falls in what is clearly not a field, then classify it as **non-cropland**;
- More than half falls in a location where it is harder to tell whether it is cropland or non-cropland, determine whether it is more likely a crop field or not a crop field, and then assign either **uncertain but likely cropland**, or **uncertain but likely non-cropland**.

2. After determining the class, if:

- The target polygon is contained entirely within a single clear class, then simply digitize a point within the center of the target box, assign the appropriate class label, and complete the assignment;
- Digitize a square polygon exactly aligned with the initial target, choose the correct class label, and then move the new polygon to the nearest location where it can be contained entirely by the assigned class.

After collecting the sample, the geometries were further refined by converting the points (sites were the target didn't have to be shifted) to polygons with the same 0.0002866° resolution, and then the full set of map reference polygons was used to extract the classified values from both the cropland probability and vectorized field boundary maps. Accuracies were assessed for the entire country, and with several zones consisting of different groupings of AOIs or agroecozones[7]

2.3.6.3 Map reference label uncertainty The size of the collected validation sample was 1207, with 1036 samples collected and interpreted by one observer (Su Ye) and 171 collected by a second observer (Estes). To evaluate the uncertainty inherent in defining the map reference labels, the pair mapped 23 common sites, showing an overall level of agreement of 87%, and a Spearman Rank Correlation of 0.76.

Although this overlap between observers was limited, the map reference classification scheme provided two additional measures of uncertainty, which were classes defined as "unsure but most likely a field" or "unsure but most likely not a field," which the reference labeller would choose when they could not with high confidence state whether the site was either cropland or not cropland. Of the 487 map reference samples that were identified as cropland, 22% fell into the lower confidence category, as did 11.5% of the 720 non-cropland samples. Across both classes, the reference labellers had lower confidence in 15.7 of sites labelled.

[7]sourced from https://wheregeospatial.com/agro-ecological-zones-ghana/

2.3.6.4 Accuracy assessment zones The zones used to assess regional variation in map accuracy are illustrated in Figure S5.

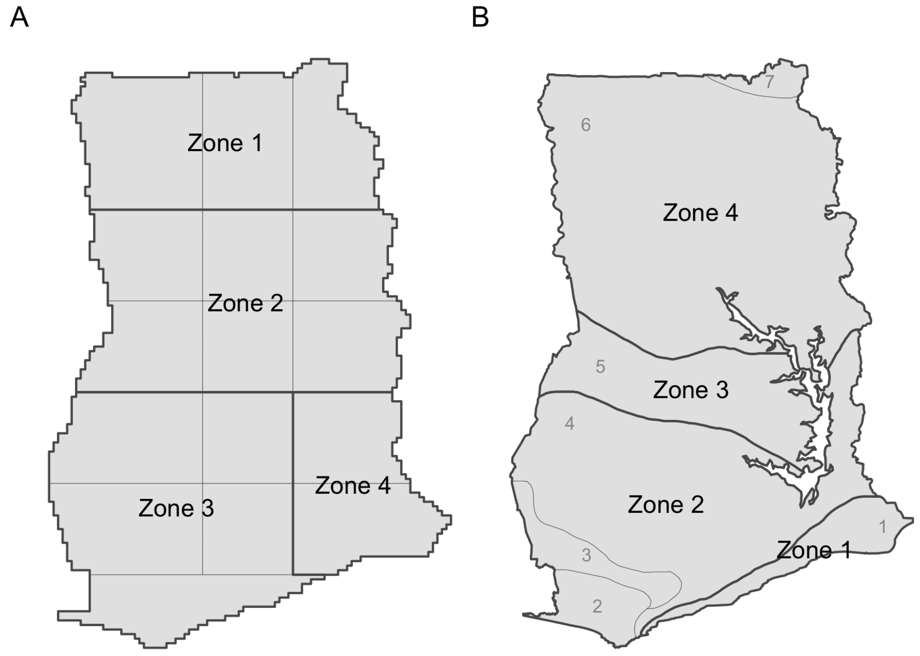

Figure S5: Four zones used to combine the A) AOIs and B) agroecozones (B), in order to assess sub-national mapping accuracy. In B, the ID number of Ghana's individual agroecozones is shown in grey: 1 = Coastal savanna; 2 = Wet evergreen; 3 = Moist evergreen; 4 = Deciduous forest; 5 = Transitional zone; 6 = Guinea savanna; 7 = Sudan savanna; 8 (not shown) = Volta Lake.

3 Supplemental Results

3.1 Image catalog and quality

The total number of PlanetScope images available for download from the Planet API (PlanetTeam 2018) per tile for each of the two major seasons is shown in Figure S6.

The image quality assessment was conducted by two separate observers (L. Song and Q. Zhang). Each observer assessed the composites for each season at all 50 of the randomly selected tiles (see main text) following the defined criteria (Table S1). To calculate the score for a given tile for each season, we summed the ranking across the four categories for each observer, rescaled the values, and then calculated the mean tile score across both observers by season. The mean difference between the two observers was -0.057 (sd = 0.081) and the mean absolute difference was 0.063 (sd = 0.076), thus one observer scored tiles about 6 percent lower than the other observer, on average.

3.2 Mapping cropland probabilities with active learning

The number of active learning iterations per AOI was three, for all but four AOIs. AOIs 10 and 14 stopped after one and two iterations, respectively, as they started with high initial validation accuracies (>83%) and showed little subsequent improvement. The models for these two AOIs were thus trained with 600 - 700 labels. AOI 15 was run for 4 iterations (900 samples), while AOI 3 underwent a second active learning cycle because the model produced during the first

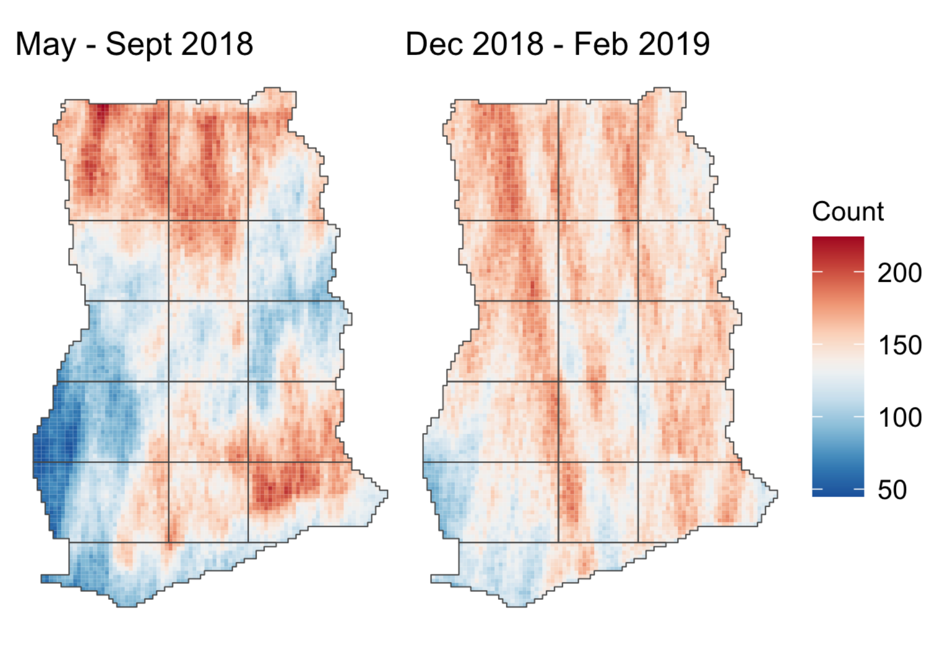

Figure S6: The density of images available through the Planet API for the five month growing season in 2018 and the subsequent three month dry season (excluding the month of November), shown in relation to the AOI boundaries.

cycle was inaccurate (see next section). In this second run, 300 initial training sites randomly selected within the AOI were used, followed by 2 subsequent active learning iterations, resulting in a training sample of 500.

3.2.1 Labelling

The locations of training, training reference, and validation points are shown in Figure S7. In AOI 3, the initial active learning cycle resulted in low accuracy because the northern part of the AOI shows low contrast between fields and the surrounding vegetation in the dry season. Training the model with the initial 500 samples resulted in large commission errors in this part of the AOI, thus we ran a second active learning cycle that began with an inital random draw of 300 training sites confined to this AOI (blue points in Figure S7A).

The distribution of training and validation sample collection effort was divided across 20 labellers, with a core group of 13 who completed more than 1,000 assignment each (Figure S8). As each training/validation task was undertaken by 4 separate labellers, 34,014 sets of labels were made. Each labeller digitized an average of 2,001 training/validation assignments.

These results include those from re-labelling the initial training in Cluster 2 (AOIs 7-9, 12, 15). This was done because we discovered a small spatial offset in the original image composites, which we corrected by reprocessing the images. We replaced the image overlays in the instances for this Cluster (Figure S7A), and the labelling team mapped these sites a second time during the production run in late 2019. The reprocessed labels were used to initially train the model for AOIs 9, 12, and 15.

3.2.2 Model performance

The differences in accuracy, AUC, and F1 between the active learning process and the random retraining at each iteration for AOIs 1, 8, and 15 (Figure S9). Small differences due to random

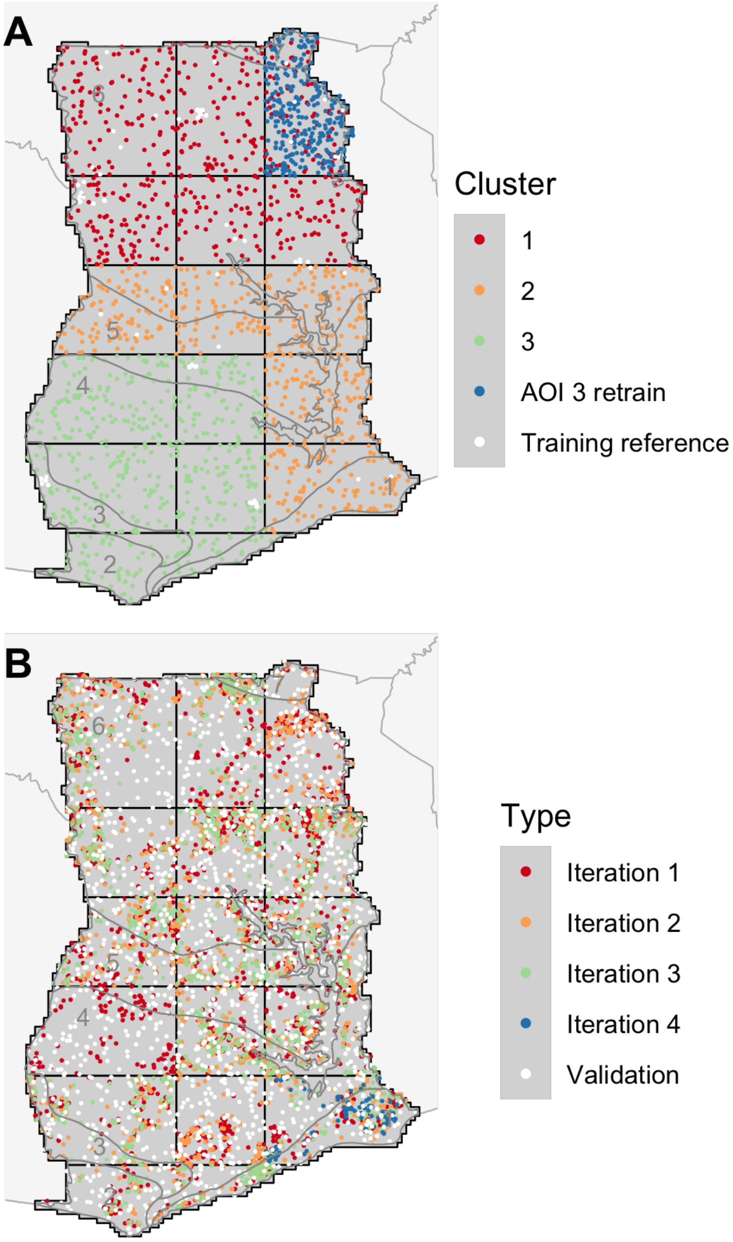

Figure S7: The distribution of A) initial randomly selected training sites, including 300 points selected to initialize a second run for retrain AOI 3, and the locations of training reference sites, and B) validation points and training sites selected during each active learning iteration. Grey background lines and show the boundaries and ID number of Ghana's agroecozones: 1 = Coastal savanna; 2 = Wet evergreen; 3 = Moist evergreen; 4 = Deciduous forest; 5 = Transitional zone; 6 = Guinea savanna; 7 = Sudan savanna; 8 (not shown) = Volta Lake.

variations in the RandomForest models are the reason for the non-zero differences at iteration 0, when both models were trained with the same level set.

The lower accuracy of actively versus randomly trained models in earlier iterations was caused by results at AOI 15, where active learning accuracy was 8.37 percent lower than random training after iteration 1 (see Figure S9). In comparison, iteration 1 active learning accuracies were 2.88 and 0.45 percent higher than random training for AOIs 1 and 8, respectively. Accuracy under active learning for AOI 15 exceeded randomized training after 4 iterations.

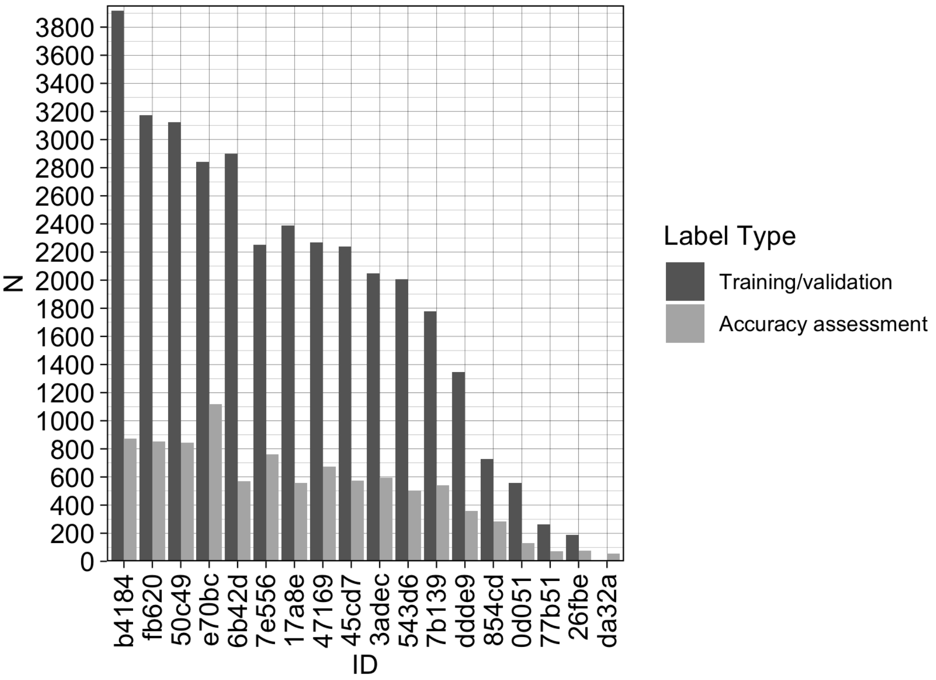

Figure S8: The A) number of training/validation and accuracy assessment assignments completed by each labeller, and B) the distributions of quality scores at training reference sites for each labeller (means indicated by X in boxplots). Labellers' identities are anonymized.

3.2.3 The impact of training data error

Two measures of label quality, average quality score per labeller and the Bayesian Risk of labels, were calculated to provide proxy measures of label error. Labeller quality was scored 9,389 times against 98 unique training reference sites, with each labeller assessed an average of 552 times at a rate of 1 training reference site for every 3.62 training site mapped. The mean of each labeller's average accuracy score was 0.71 (range 0.6 to 0.85; Figure S10).

The average Bayesian Risk of each training and validation site is shown in Figure S11, and the distribution of risk values per AOI and the initial training clusters in Figure S12. The three initial clusters include the second mapping of Cluster 2 (Figure S7A).

The average Bayesian risk was 0.122 for training labels and 0.127 for validation labels. Risk was highest in the northern AOIs (AOIs 1-6; Figures S11-S12), falling between 0.157 for training and 0.173 for validation labels, and lowest in the southwestern AOIs (AOIs 10, 11, 13, 14, 16; training risk = 0.079; validation risk = 0.065). Label risk in the central-southeastern AOIs (AOIs 7-9, 12, 15) was slightly lower (training = 0.127; validation = 0.136) than in the north. Labeller experience also appeared to reduce risk, which we observed during a relabelling of the 500 initial random site in this cluster; the mean risk of the updated labels was 0.055, compared to 0.172 for original labels.

Probability images resulting from Random Forests models trained with labels generated under three different labeling strategies are illustrated in Figure S13. These included consensus labels, and those individual labels that were likely to be the most and least accurate for each training site. Label accuracy was based on the mean score of each labeller against the training reference sites (Figure S10), as assessed when labelling a given AOI. These images were created for a single tile in AOI 1.

The association between the average label risk per AOI and several model performance metrics,

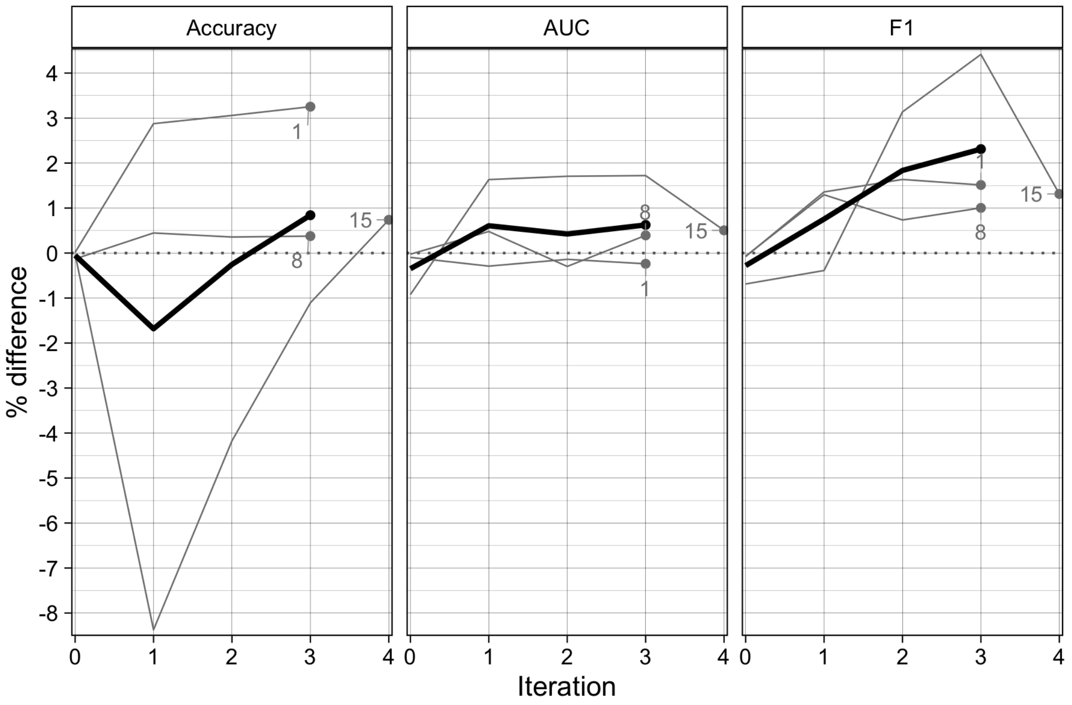

Figure S9: The percent difference in performance metrics per iteration for AOIs 1, 8, and 15 (grey lines with numbers indicating AOI; the black line indicates average difference across the three AOIs) when comparing models trained using active learning versus those trained using randomly selected sites. Postive percentages indicate superior performance by active learning, negative percentages the inverse.

assessed using Spearman Rank Correlation, is shown in Table S3.

Table S3: Spearman's Rank correlation between the average label risk per AOI and a variety of model performance metrics.

	Accuracy	AUC	F1	Precision	Recall	FPR
r	-0.824	-0.568	0.456	0.629	0.206	0.688

3.3 Map accuracy

3.3.1 Categorical accuracy

In addition to the map accuracies and area estimates calculated per AOI zone (reported in main text; see Figure S5A), the accuracies were also assessed within several different groupings of agroecozones (Figures S5B and Table S4).

3.3.2 Field area and number

To mean, median, and distributions of the average area of segmented field boundaries over the 100 validation sites in each AOI are compared to the areas of the polygons digitized by the most accurate labeller over the same sites in Figure S14. The same statistics for average number of segments versus average number of labelled polygons across validation sites in each AOI are shown in Figure S15.

Table S4: Map accuracies and adjusted area estimates for the 3 m pixel-wise classifications (based on RandomForest predictions). Results are provided for four different groupings of Ghana's 8 agroecozones zones (Zone 1 = Coastal savanna; Zone 2 = Wet evergreen, Moist evergreen, and Deciduous forest; Zone 3 = Transitional zone; Zone 4 = Guinea savanna and Sudan savanna) plus the entire country. The error matrix (with reference values in columns) provides the areal percentage for each cell, and the producer's (P), user's (U), and overall (O) map accuracies and their margins of error (in parenthesis) are provided, as well as the sample-adjusted area estimates (in km^2) and margins of error.

		**Non-crop**	**Crop**	**Total**	**U**	**O**	**n**	**Area**
	Non-crop	65.4	5	70.4	92.9 (9.7)	82.9 (7.7)	28	7985 (797)
	Crop	12	17.6	29.6	59.4 (12.1)		64	2332 (797)
Zone 1	P	84.5 (9.7)	77.8 (12.1)					
	n	52	40					
	Non-crop	69.5	11.6	81	85.7 (4.7)	82.4 (4.3)	217	33693 (1918)
	Crop	6	13	19	68.5 (10.7)		73	10974 (1918)
Zone 2	P	92.1 (4.7)	52.9 (10.7)					
	n	209	81					
	Non-crop	87.4	9	96.4	90.7 (8.8)	89.9 (8.5)	43	59112 (5653)
	Crop	1.2	2.4	3.6	67.5 (8.2)		126	7596 (5653)
Zone 3	P	98.7 (8.8)	21.2 (8.2)					
	n	80	89					
	Non-crop	70.4	7.9	78.3	89.9 (3.7)	85.6 (3.1)	258	86104 (3420)
	Crop	6.5	15.2	21.7	70.0 (4.7)		370	25802 (3420)
Zone 4	P	91.6 (3.7)	65.8 (4.7)					
	n	343	285					

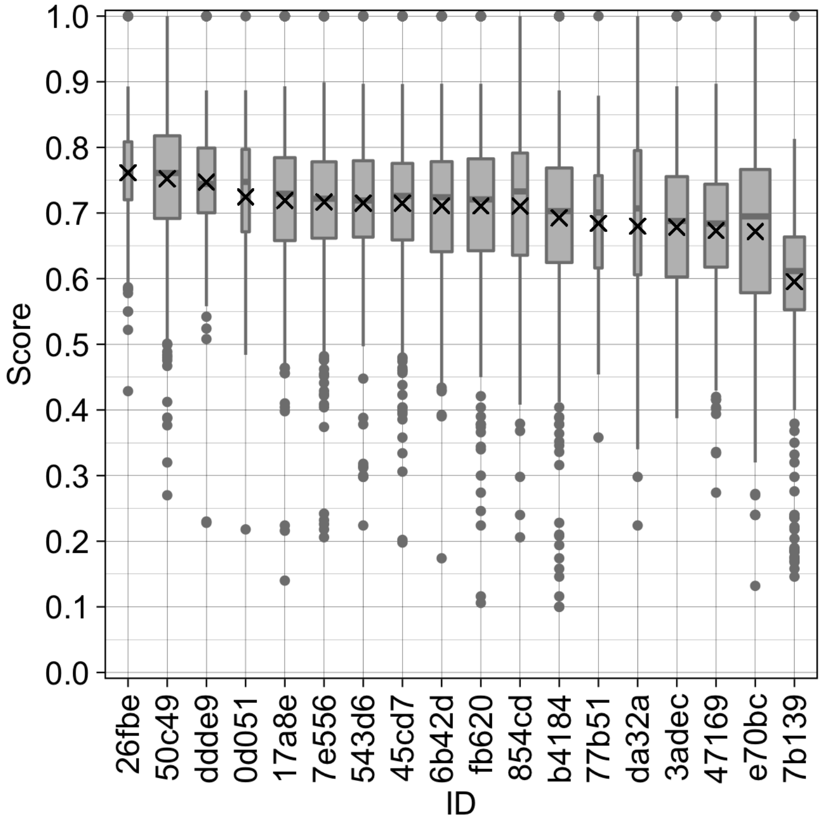

Figure S10: Boxplots showing the distributions of quality scores from the accuracy assessment assignments undertaken by each labeller (means indicated by X in boxplots). Labellers' identities are anonymized.

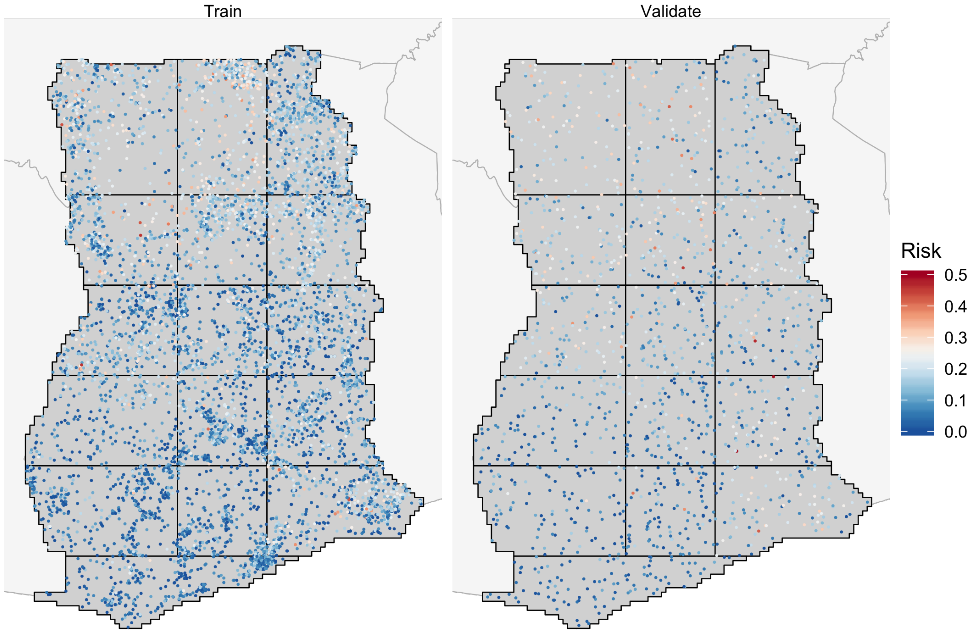

Figure S11: The average Bayes Risk of each training and validation site in Ghana.

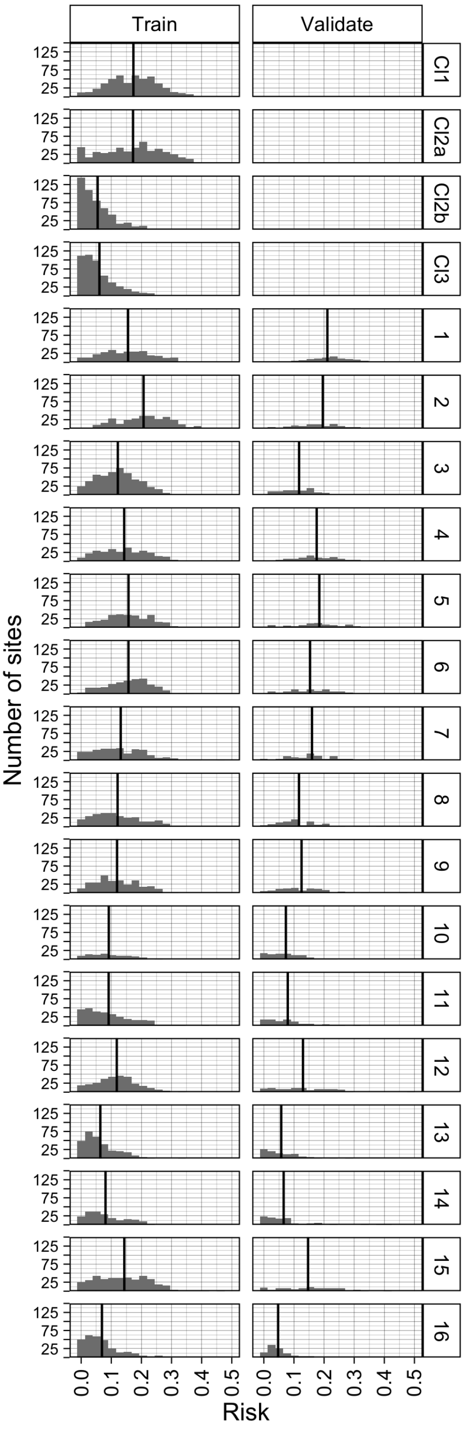

Figure S12: The distribution of Bayesian Risk values for training (left column) and validation (right column) sites in each AOI, with the average value indicated by vertical lines. The first three rows (panel titles beginning with 'Cl') indicate distributions of Bayes Risk in the initial training clusters, including the first ('Cl2a') and second ('Cl2b') mappings of Cluster 2.

3.4 Cropland characteristics

3.4.1 Field area and number

To examine average field sizes, and the total number, the mean segment size per 0.05 degree tiles was calculated and mapped, as well as the total number of fields per tile (Figure S16).

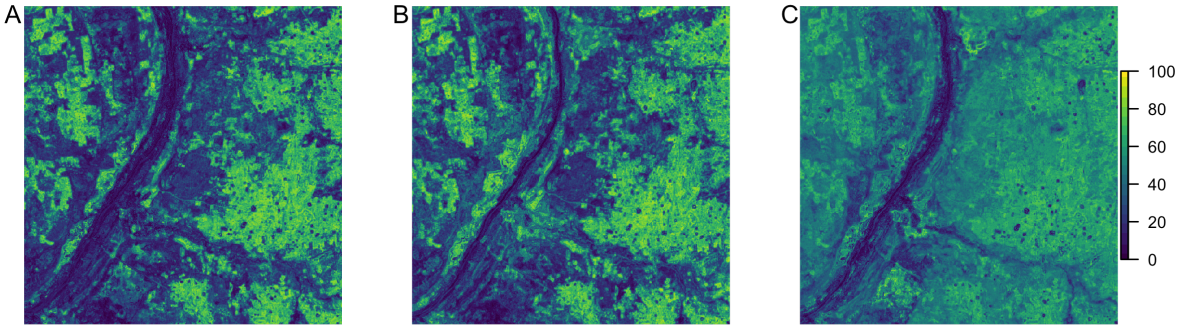

Figure S13: Cropland probability images produced by Random Forests models trained with A) consensus labels, B) the most accurate individual labels, and C) the least accurate individual labels.

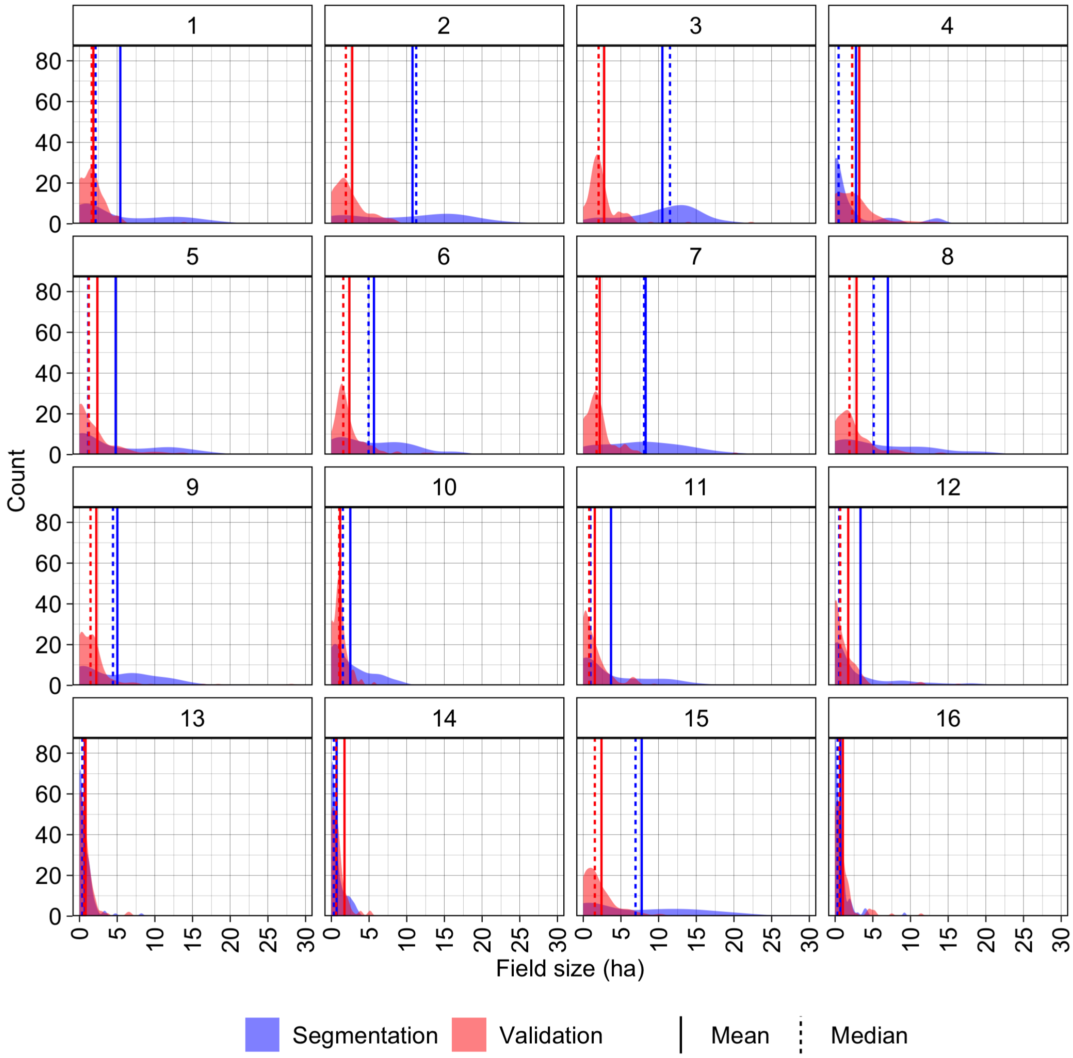

Figure S14: The distributions of the average areas (in hectares) of segmented field boundaries (shown in blue) at the 100 validation sites per AOI, compared with the average areas of field boundaries digitized (shown in red) by the most accurate worker to label each site. Vertical lines indicate the mean and median of each distribution.

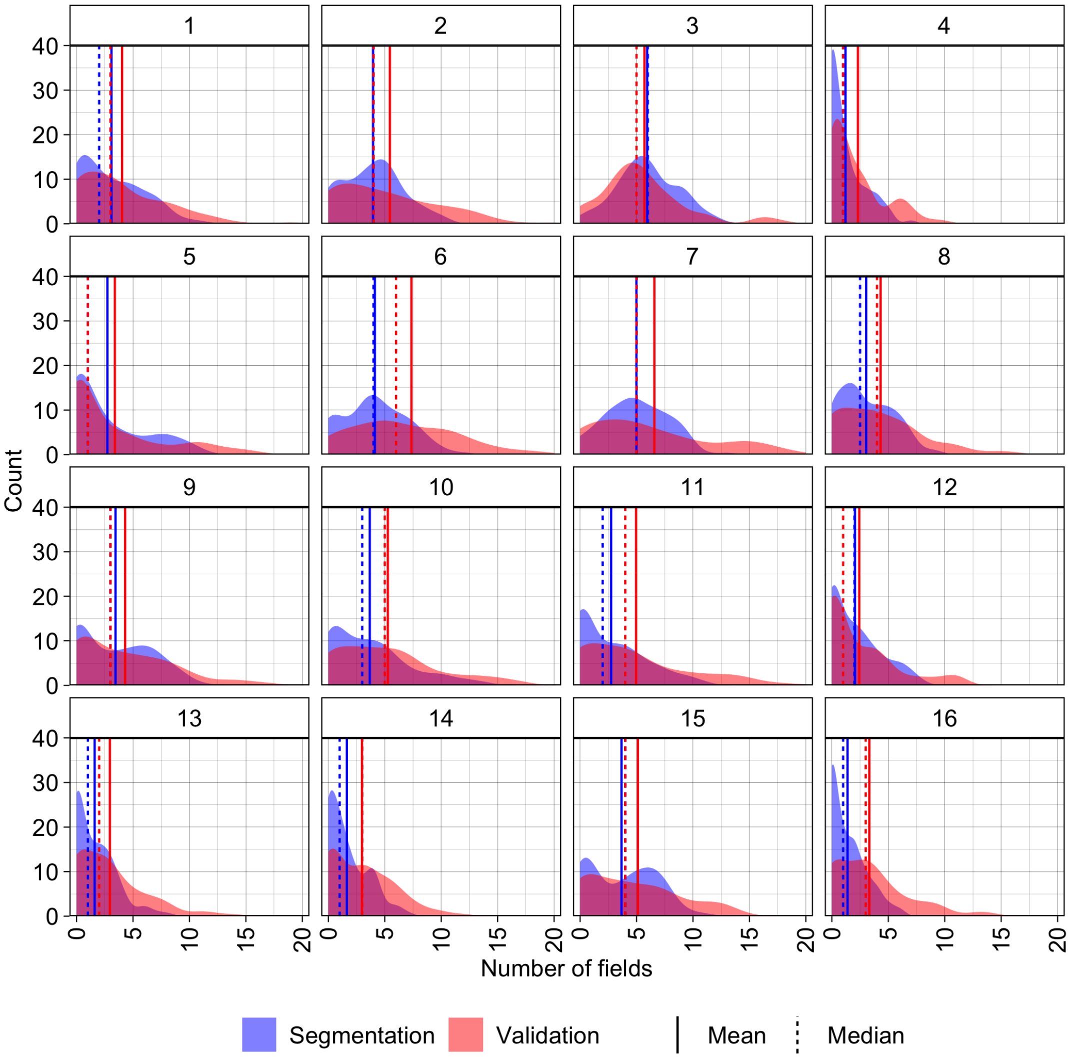

Figure S15: The distributions of the average number of segmented field boundaries (shown in blue) at the 100 validation sites per AOI, compared with the average number of digitized polygons (shown in red) by the most accurate worker to label each site. The mean and median of each distribution is shown. Vertical lines indicate the mean and median of each distribution.

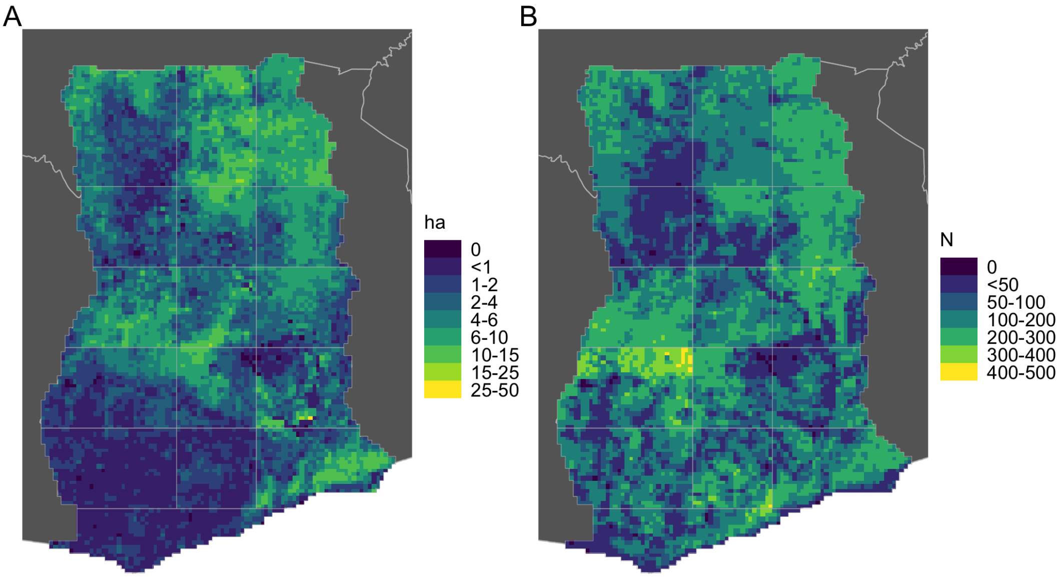

Figure S16: The A) average sizes of fields and B) total number of fields in each 0.05 degree tile, as calculated from the vectorized field boundaries.

4 References

Azavea. 2020. Raster Foundry. https://github.com/raster-foundry/raster-foundry.

Hijmans, R. J. 2020. Raster: Geographic data analysis and modeling. Manual.

Pebesma, E. 2018. Simple features for r: Standardized support for spatial vector data. The R Journal 10:439–446.

PlanetTeam. 2018. Planet application program interface: In space for life on Earth. https://api.planet.com, San Francisco, CA.

Stehman, S. V., and G. M. Foody. 2019. Key issues in rigorous accuracy assessment of land cover products. Remote Sensing of Environment 231:111199.
